# Health System Response to Refugees’ and Migrants’ Health in Iran: A Strengths, Weaknesses, Opportunities, and Threats Analysis and Policy Recommendations

**DOI:** 10.3389/ijph.2023.1606268

**Published:** 2023-09-28

**Authors:** Ahad Bakhtiari, Amirhossein Takian, Alireza Olyaeemanesh, Masoud Behzadifar, Afsaneh Takbiri, Saharnaz Sazgarnejad, Sahar Kargar

**Affiliations:** ^1^ Health Equity Research Centre, Tehran University of Medical Sciences, Tehran, Iran; ^2^ Department of Health Economics and Management, School of Public Health, Tehran University of Medical Sciences, Tehran, Iran; ^3^ Department of Global Health and Public Policy, School of Public Health, Tehran University of Medical Sciences, Tehran, Iran; ^4^ Social Determinants of Health Research Center, Lorestan University of Medical Sciences, Lorestan, Iran; ^5^ Department of Public Health, School of Health, Sabzevar University of Medical Sciences, Sabzevar, Iran; ^6^ School of Medicine, Tehran University of Medical Sciences, Tehran, Iran

**Keywords:** migrant and refugee health, healthcare access, SWOT analysis, health systems, Iran

## Abstract

**Objective:** Iran is one of the main hosts of Afghan refugees. This study aims to provide comprehensive evidence to increase Afghan migrants’ access to healthcare services in Iran.

**Methods:** To assess the health system’s response to Afghan migrants in Iran, we conducted three phases for SWOT analysis, including: 1-developing a review and comprehensive analysis of documents, laws, and, programs, 2-conducting semi-structured interviews with policymakers and experts, and 3-mapping the results through the Levesque’s conceptual framework for healthcare access.

**Results:** We evaluated the response of the health system to Afghan migrants’ health needs in three domains: 1-Approachability and ability to perceive migrants; 2-Ability to reach, engage, and availability and accommodation and appropriateness; 3-The ability to pay and affordability. For each of the three domains, we identified strengths, weaknesses, opportunities, and threats, complemented with evidence-based suggestions to improve migrants’ access to needed healthcare services.

**Conclusion:** Given the rising trend of immigration and deteriorating financial crises, we recommend appropriate strategies for the adoption of specialized focus services, gateway services, and restricted services. Also simplifying financial procedures, and implementing innovative insurance mechanisms are essential.

## Introduction

The United Nations (UN) defines a refugee as “someone who changes his or her country of usual residence, regardless of the reason for migration or legal status” and a migrant as “any person who is outside a State of which they are a citizen or national, or, in the case of a stateless person, their State of birth or habitual residence.” This definition does not include the hardships of this type of residential change. Refugees stand among the most vulnerable people, whose numbers are on the rise [1]. According to the UN Refugees Agency’s estimations in 2023, as a result of harassment, conflict, violence, human rights violations, or other events that disrupted public order, 108.4 million people will be relocated around the world, 35.3 million of whom were classified as Refugees, 5.4 million as Asylum seekers, 62.5 million as Internally displaced people (IDPs), and 5.2 million other people in need of international protection [2]. Just three countries account for more than half of the world’s refugees and other individuals in need of international protection; (Syrian Arab Republic: 6.8 million, Ukraine 5.7 million, Afghanistan 5.7 million of which 3,400,000 are in Iran) [3, 4]. In line with the previous year, 56% of all persons evacuated across borders resided in only 10 countries; Turkey, Pakistan, and Iran are the top three on this list. In this paper, our main focus will be on Afghan refugees currently residing in Iran [2].

Afghan refugees’ registrations began in 1979 in Iran, peaked in the 1990s (3 million), and remained stable until 2004 (around 1 million). According to the 2016–17 National Population and Housing Census, 1,654,388 foreign nationals lived in 31 provinces across Iran, of whom 1,583,979 were of Afghan origin. Although it is estimated that 8 million Afghans live in Iran, the majority of whom are undocumented and therefore are not counted in the national census [5].

The main health problems of Afghan migrants in Iran are non-communicable diseases (NCDs), communicable diseases, food insecurity and malnutrition, Low immunization coverage, and Psychological disorders [6]. Although healthcare facilities in Iran are available to provide healthcare services to Afghan migrants for any given illness, as most of them are uninsured, affordability to pay is a big challenge that has led to deteriorating their health status over time.

Various countries have developed four care models to serve refugee and migrant populations that include mainstream services, specialized-focus services, gateway services, and limited services [5]. Since the introduction of the health transformation plan (HTP) to reach a universal public health insurance program in 2015, Iran has adopted a mainstream model of care to serve its refugee and migrant population, allowing all UNHCR-registered refugees living in the Islamic Republic of Iran to access the same level of health services as Iranian citizens. Refugees can sign up for the program at the local government offices and receive care at government hospitals and clinics. The Iranian government, UNHCR, and other donors are all contributing to the program. UNHCR covers the costs of premiums for the most vulnerable refugees, while other refugees must pay their premiums.

Barriers obstruct the process of receiving healthcare in a variety of ways. The Levesque conceptual framework for healthcare access provides policymakers with a clear perspective of the effect areas by including characteristics such as perception of health needs and desire to care, healthcare seeking, healthcare reaching, and healthcare utilization [7]. The literature identified the most important barrier for migrants to receive health services is their ability to pay [7, 8]. Various characteristics could affect migrants’ ability to pay, i.e., the host country’s labor laws, migrants’ insurance coverage, migrants’ income, type of employment, and tax legislation.

This study aims to provide evidence to increase migrants’ access to healthcare services. We conducted a SWOT (Strengths, Weaknesses, Opportunities, and Threats) analysis of Iran’s health system to develop access to healthcare services based on Levesque’s conceptual framework, followed by recommendations for the Iranian health system.

## Methods

### Study Design

SWOT Analysis is a strategic planning tool to identify the strengths, weaknesses, opportunities, and threats present in a project, or any other scenario that requires a decision (organization or program). Internal and external elements and existing and future possibilities are all evaluated in a SWOT analysis [9]. SWOTs are defined using the following criteria:

• Strengths are internal organizational characteristics that aid in the achievement of the goal.
• Weaknesses are internal organizational characteristics that obstruct the achievement of the goal.
• External factors that aid in attaining the goal are referred to as opportunities.
• External conditions that are adverse to achieving the goal are referred to as threats [9].

According to the Revised SWOT analysis, which is developed for healthcare organizations; Stakeholder expectations, resources, and contextual factors should all be considered [10]. We designed and conducted three phases to complete the SWOT analysis of Iran’s health system response to Afghan migrants.

**Phase 1** was conducted in two steps, as follows:

I. Investigating Iran’s background in healthcare planning for Afghan migrants (including content analysis of documents, laws, and programs),
II. Identifying SWOT items and policy recommendations by conducting semi-structured interviews with policymakers, administrators, migrants, and experts;

#### Data Collection

This is a qualitative study. We collected data through a comprehensive review of laws, regulations, and associated documents related to foreign nationals in the legal systems of Iran, followed by semi-structured, face-to-face, in-depth interviews.

### Comprehensive Review of Laws, Regulations, and Associated Documents

To provide a comprehensive literature review of laws and other related documents, the National Database of Parliamentary Laws and Regulations [11], the government [12], and the Ministry of Health and Medical Education (MoHME) [13] were searched using terms: foreign nationals, refugees, immigrants, or asylum seekers and health, healthcare, treatment, employment, or insurance. These databases contain all laws and regulations passed by the government, parliament, and ministries as well as a history of laws, back since 1906. In addition, we searched the websites of the MoHME, the Iranian Health Insurance Organization (IHIO), the Bureau for Aliens and Foreign Immigrants Affairs website (BAFIA), and other related organizations. To ensure inclusivity and identification of all related materials, two researchers worked independently on the inquiry.

### Semi-Structured Interviews

We conducted 76 semi-structured, face-to-face, in-depth interviews with a purposively selected diverse group of participants including government administrators, Afghan (documented and undocumented), MoHME officials, healthcare providers, representatives from insurance organizations, employers of Afghan in Iran, NGOs, and academics, with the experience of participating in the planning, decision-making, and support of migrant healthcare programs (See Table 1 for details). The interview guide was developed using Levesque’s conceptual framework for healthcare access. We assured the interviewees of anonymity and confidentiality and obtained their written or verbal consent before the interview. Interviews lasted 18–80 min (interviews with refugees took less time), digitally recorded and transcribed verbatim. We also took notes during the interview and sought feedback from selected interviewees and held several meetings for data interpretation.

**TABLE 1:** The characteristics of interviewees (Iran, 2023).

	Type of interviewees, expertise		Number
1	MoHME	Managers and experts of related departments	9
2	State Walfare Organization of Iran	Expert in migrant affairs	1
3	Rehabilitation center	Expert in migrant affairs	1
4	UNHCR	Expert in migrant affairs	2
5	BAFIA	Expert in migrant affairs	1
6	NGOs		21
7	IHIO	Expert in migrant affairs	1
8	Asia and Alborz insurance	Expert in migrant affairs	2
9	Social Security Organization	Expert in migrant affairs	3
10	Health services provider (Hospitals, health houses, and health posts)	Manager, physician, nurse, PHC staff	20
11	Afghan migrants	Legal and illegal migrants, with insurance, without insurance	11
12	Other	hospital charity	4

#### Data Analysis

We used MAXQDA Version 10 software for data management and content analysis of documents and interviews. Three researchers carried out the analysis of interviews and related documents using the framework analysis approach. We considered the Levesque conceptual framework for healthcare access as the thematic framework. Differences in coding were discussed to establish consensus, sub-themes were found, a hierarchy of topics was constructed, and a general coding and framework (see Supplementary Appendix SA for further detail). We developed a preliminary list of SWOT and recommendation items based on the findings in Supplementary Appendix SA.

**Phase 2**: Best experiences and recommendations based on a scoping review.

We conducted a scoping review to identify and understand the best experiences and recommendations to provide optimal responses of health systems to the health needs of migrants around the world. The following five steps were carried out:

Step one: Study Questions;
Step two: Identification of relevant studies; (Study time frame, databases, and websites, the search terms);
Step three: Selection of included studies;
Steps four and five: charting data; organizing, summarizing, and reporting the results [14].

Detailed information on each step and related methods and results is presented in Supplementary Appendix SB.

**Phase 3**: Mapping the result of the scoping review and SWOT based on Levesque’s conceptual framework for healthcare access.

In the final phase, three researchers independently categorized the results of the first two phases into the structures of SWOT dimensions and Levesque’s conceptual framework [15, 16]. The framework presents a comprehensive assessment of healthcare access, which includes variables such as approachability, acceptability, availability/accommodation, affordability, and appropriateness. It takes into account the socioeconomic characteristics of the community to include the five comparable capacities of individuals and populations to recognize, seek, reach, pay for, and engage in healthcare. Levesque’s conceptual framework was selected as one of the most comprehensive models because it takes into account various social, cultural, and economic dimensions as a series of processes of access to health services, as well as the viewpoint of the provider, the patient, policymakers, and health managers.

**FIGURE 1:**
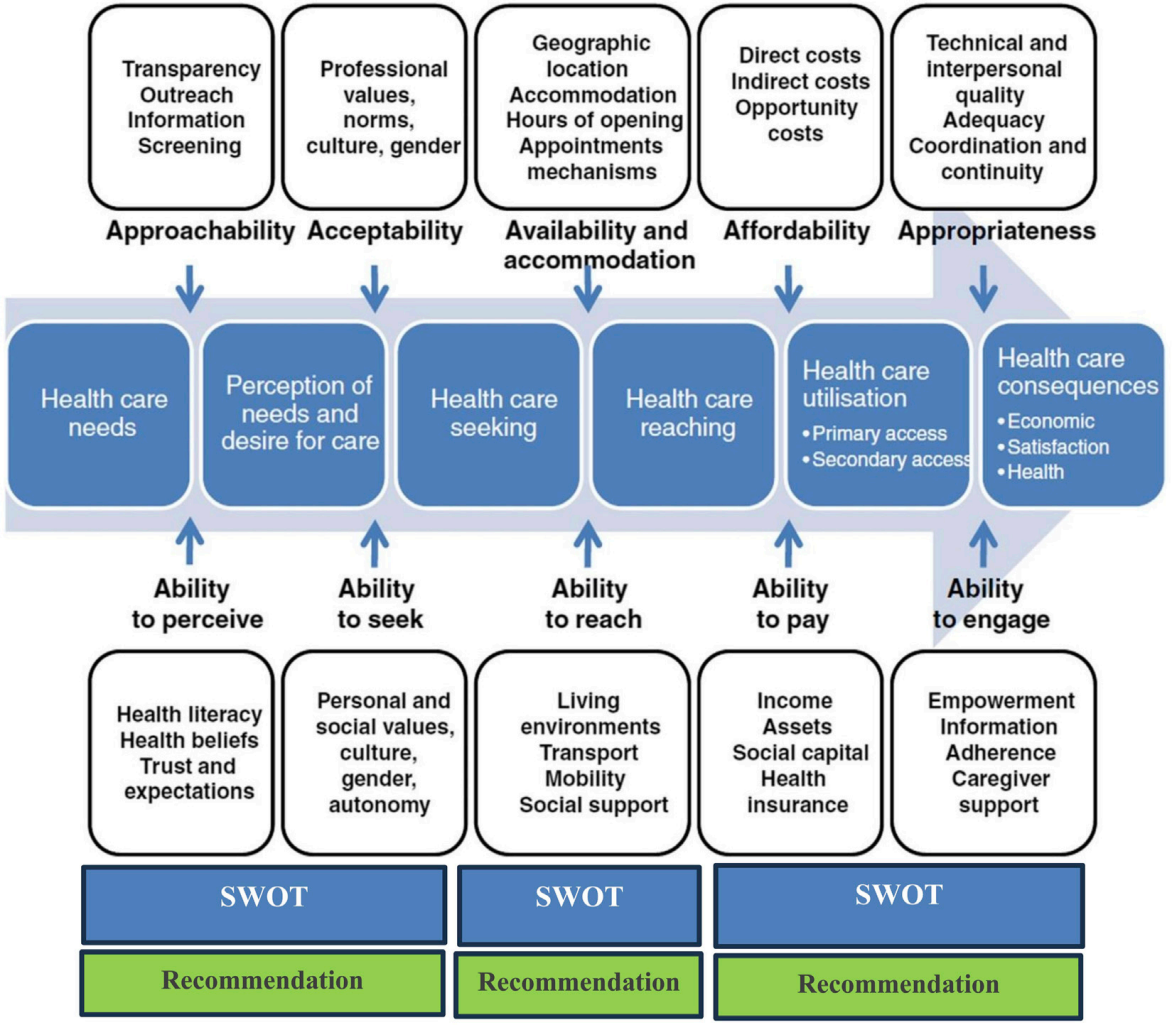
Levesque’s conceptual framework for healthcare access [16, 17] (Iran, 2023). Source: Cu et al. Redesigned by authors.

To address the disagreements in the categories, the results were unified, and the conflicting cases were discussed by all members of the study team. Thus, each component of the framework contains a SWOT analysis based on Iran’s situation (Figure 1).

## Results

Below, are the five categories in which the content of the documents and experts’ opinions were analyzed:

❖ A.1 Iran’s accession to international conventions on refugees;
❖ A.2 Migrants’ Healthcare rights in Iran;
❖ A.3 Migrants’ right for access to healthcare services in Iran;
❖ A.4 Health insurance schemes for migrants in Iran;
❖ A.5 Health services and financial protection for migrants; barriers and challenges in the Islamic Republic of Iran;

Supplementary Appendix SA provides more detail about phase one of Iran’s situation. In phase two**;** following the initial search, we found 1,590 articles, 254 of which were removed as duplicates. We reviewed the titles and abstracts of 1,336 articles and excluded 1,155 articles due to lack of relevance to the current study. Finally, 34 studies were selected based on the study criteria. Supplementary Appendix SB presents a set of countries’ experiences and recommendations for migrant health.

We considered all dimensions and complexities of healthcare access to address all related issues. Levesque’s Conceptual Framework of Access to Health, published in 2013, investigates the five dimensions of access as well as the five abilities of the population to access healthcare. Tables 2–4 present the details of global recommendations as well as an analysis of Iran’s situation in each of Levesque’s dimensions.

**TABLE 2:** Strengths, Weaknesses, Opportunities, and Threats and recommendation for approachability and ability to perceive [15, 18–21] (Iran, 2023).

	SWOT		Recommendation based scoping review and interview	Details
Approachability and acceptability, Ability to perceive and ability to seek	S	1. Iran's healthcare system recognizes the documented migrant population's entitlement to healthcare access, and there is no legal restriction on undocumented migrants using healthcare services (although there are other obstacles). 2. Providing healthcare in accordance with cultural and Islamic principles 3. Integrating respect for cultural and social values and patient rights education into the curriculum of many fields 4. Depending on religious and cultural beliefs, both male and female providers are available. 5. Using PHC services does not require a legal certificate	• 1951 Geneva Convention and 1967 Protocol social rights apply to refugees. (T 16, 17, 18, 19, 20) • In the form of a prioritized package, migrants should receive all levels of healthcare preventative, health promotion, diagnostics, curative, and rehabilitation. (W 6) • There are four issues for newly arrived migrants that health service providers should consider: ○ Assessing the current health condition. (W 9) ○ Health risk assessment. (W 8) ○ Providing information about the healthcare system of the host country (W7) and ○ Health education. (W 4, T 1, 2, 9) • Increasing awareness of schemes and benefits ○ Awareness campaigns through advertisements in the media (W 1, 2, 3. T 10) ○ Awareness campaigns targeted specific places (W 1, 2, 3, 9) • Improving the management and organization of insurance programs: ○ Improving the information system ○ Staff training (T 15) ○ Transparent management • Acceptability ○ For services that are culturally appropriate by addressing linguistic or cultural barriers, primary healthcare can be tailored to the needs of a specific vulnerable group. ○ A health worker in the community: A layperson, who is a trusted member of the community or has a thorough understanding of it, serves as a frontline worker who helps community members overcome cultural and linguistic barriers and gain access to primary healthcare. (W 5) ○ Group visits Rather than providing individual care, primary healthcare is provided to a group of people with similar vulnerabilities or conditions. (W 8) • Approachability ○ Proactive identification of need: A mechanism is put in place to proactively identify vulnerable patients’ needs for primary healthcare and provide additional support to avoid the negative consequences of unmet needs. (W 6, 7, 8) ○ Information and navigation: A service that informs and supports individuals on where, when, and how to obtain primary healthcare. (W 3, 7) ○ The brokerage of primary healthcare services: A service that assists vulnerable patients in connecting with a primary care provider or primary healthcare service, including single entry points to access and priority queueing based on vulnerability indicators. (W 6, 8) ○ Provision of primary healthcare services to the general public to reach vulnerable populations; primary healthcare services should be extended beyond the physical limits of primary care settings. (W 6) ○ Inter-organizational/inter-sectoral care pathways: Primary healthcare organizations collaborate with other organizations (both within and outside the health system) to develop procedures that ensure vulnerable groups have timely access to needed services. ○ Making proactive appointments and maintaining proactive contact: Appointment-making processes in primary care that draw vulnerable patients in for care and keep in touch with them. (W 8) • In certain countries (e.g. Turkey), refugees are better protected since they are enabled to migrate from the informal to the formal economy. Refugees being included in contributing schemes can assist in avoiding labor market distortions. Without paying social security contributions (and typically lower wages), employers may hire refugees, causing labor market inefficiencies as well as host-refugee conflicts. (T 4, 12,13, 14, 21, 22, 23) • Establishing international health-focused communication channels for migrants (W 1,2,3,) • Migrants’ attitudes and cultures about health and insurance should be strengthened through health education. (T 12) • Providing families who enroll in insurance programs with economic and social benefits. (T 4, 6, 13, 14) • Utilizing the primary insurance programs of the country where migrants are living will boost their confidence in insurance providers. (T 3, 7) • Issuing identifying documents to migrants who do not have documents for a variety of reasons. (For the procedure of receiving medical care) (T 16, 17, 18, 19, 20)	-Appendix A. Sections A.1. & A.2, A.3, A.5.1 and related quotations, & A5.2 and related quotations. -Appendix B
	W	1. Lack of awareness of the health insurance plan among Afghan migrants 2. Providing inadequate information to the migrant community about their rights, options, and health facilities 3. The methods used to inform migrants are not clear and regular. 4. Insufficient focus on public health education initiatives that are tailored to migrants' needs and their linguistic and cultural aspects. 5. Poor utilization of the capacity of the health volunteers to promote migrants' health 6. Treatment-oriented approach to migrant insurance coverage. 7. Lack of familiarity with healthcare providers among migrants 8. Late visits by migrants to health facilities (referring in the acute stages of the disease) 9. Men use PHC facilities less frequently.		
	O	1. Widespread Internet and media coverage to inform and teach new arrivals 2. The concentration of migrants in specific geographical areas and joined society 3. The government can give work licenses to foreign nationals, including refugees, under Article 122 of the Iranian Labor Law 4. Convention relating to the Status of Refugees of 28 July 1951, and the Protocol on the Status of Refugees of 31 January 1967 5. Convention No. 19 on Equal Treatment of Domestic and Foreign Workers in Compensation for Work-Related Accidents (1925) 6. Social Security Act (1975) 7. The Iranian government ratified the 1951 Refugee Convention and its Protocol (1967) on July 28, 1976, with the exception of Articles 17 (Wages on Employment), 23 (Government Charity), 24 (Labor and Social Insurance Laws), and 26 (Wages on Employment) (Freedom In commuting). 8. Afghan migrants who volunteered to engage in projects including health 9. Language and cultural affinities between Afghan migrants and Iranian society 10. Rather than putting migrants in camps and isolating them, integrate them into society 11. Education of Afghan students in Iran, regardless of whether they are living there legally or illegally		
	T	1. Migrants' low level of health literacy 2. The low-grade state of the healthcare system in migrants' countries of origin and the health needs that are not initially understood 3. unfavorable disparities in health attitudes (not believing in paying insurance premiums, maternity visits) 4. Low economic and social status of migrant 5. Temporary employment in the informal economy is a common occupation among non-skilled refugees. 6. Marginalization 7. lack of understanding of insurance benefits among migrants 8. The family's father usually makes economic decisions in Afghan and Iranian 9. self-medication, and traditional therapies 10. The absence of popular and specialized media that migrants could use to promote health views 11. Migrants had no desire to register the insurance due to a lack of trust in the insurance company. 12. Employers' discriminatory attitudes towards migrants led them to avoid paying premiums 13. Employers of Afghan migrants hire foreign nationals to avoid paying premiums. 14. Absence of accountability process for offending employers 15. negative opinions about migrants 16. The lack of a birth certificate and an accurate date of birth made it difficult to provide Afghans with insurance and benefits such as retirement 17. Due to the lack of an official marriage certificate, the spouse’s insurance coverage was jeopardized 18. Lack of legal residency was an obstacle to receiving insurance coverage by the Social Security Administration. 19. The issuance of a work card was reported as one of the barriers to accessing insurance 20. The lack of official employment permits for Afghan women has led to the non-issuance of work cards for them and, as a result, the lack of insurance coverage for working women. 21. Migrants working in dangerous and laborious tasks 22. lack of education to develop the skills needed to earn more income		

**TABLE 3:** Strengths, Weaknesses, Opportunities, and Threats and recommendation for the ability to reach and availability [22] (Iran, 2023).

	SWOT		Recommendation based scoping review and interview	Details
Ability to Reach and Availability and Accommodation & Ability to engage and appropriateness	S	1. The existence of a coherent PHC network, especially in marginal and remote areas 2. Comprehensive health centers in urban and rural settings as a part of the country's primary health care system (jointly used by Iranians and migrants) providing health services to migrants 3. Each PHC center's population and geographic area are defined. 4. Coexistence of male and female health professionals 5. The existence of a nutritionist and a psychologist in PHC facilities 6. Free delivery of a variety of services to migrants, including tuberculosis diagnosis and treatment 7. Using mainstream services as a model for delivering services to migrants 8. Sanitary facilities of guest cities (camps) are available 9. Migrants (including refugees, passport holders, and undocumented migrants), like Iranian citizens, have access to PHC services. 10. No waiting line to receive health services in Iran	• The ultimate goal of providing healthcare to refugees is integrating them into the host country's national health system. (Mainstream services). Employing specialized units to provide dedicated refugee health services is a strategy used by health systems to deliver health services in the early years of migrant arrival. These units are the channel for migrants to enter the health system of the host countries, and the existence of these centers has the following advantages: (W 1, 2, 3, 5, 6, 7, 8. T 1) ○ Their human resources for health usually receive fixed salaries ○ Volunteer human resources are used in them in a coherent manner ○ Their healthcare workforce is familiar with the culture and health problems of migrants ○ Foreign nationals experience fewer language, cultural, and service delivery problems ○ Foreign nationals in these centers get familiarized with the health system of the host country ○ These centers train migrants to enter the host country's public health system. • Improving healthcare delivery ○ (a) Improving the health care package ○ (b)Controlling the price of services ○ (c) Improving the quality of services to attract more of the eligible population ○ (d) Modifying enrolment ⁃ Simplifying the enrolment procedure (W 4) • Availability & Accommodation ○ Longer working hours to meet the needs of vulnerable populations: A primary healthcare organization extends its operating hours beyond 9 a.m. to 5 p.m. (W 9) ○ Access to advanced features: A scheduling system that provides urgent care by a known primary healthcare team, triggers planned appointments when necessary, and allows patients to schedule an appointment at the most convenient time. ○ Health-related virtual services: For consultations or monitoring health conditions, use videoconferencing, phone, email, text message, apps, and so on. (W 7, 8, 9) ○ Services that are available on-demand: Patients who come in without an appointment can receive services. ○ Transportation services: Organizing transportation for patients who have difficulty getting to primary care facilities. ○ Task shifting or role expansion Upskilling: A healthcare worker who works with vulnerable patients on a regular basis to improve workforce capabilities. It's possible that formal providers' scope of practice will be expanded or that laypeople will be trained. (W 6, 8) ○ All-in-one solution: At the point of contact, multiple health and social services are provided in one location to provide comprehensive care to hard-to-reach vulnerable patients with complex needs.	-Appendix A. Sections A.3, A.4, A.5.3 and related quotations. -Appendix B
	W	1. Inequitable distribution of hospitals and clinics at the second and third-level 2. Due to the imbalance between the quantity of staff and the population served, PHC centers have a high workload. 3. Early on in migrant arrival, there is a lack of utilization of specialist units to offer dedicated refugee health services. (Requires a shift to Mixed models of care) 4. The limited registration period for health insurance 5. Lack of initial comprehensive health assessment or “welcome visit” to new migrants 6. Per capita shortage of doctors and nurses compared to countries in the region 7. Low hospital beds per capita, especially for marginalized areas 8. The low number of nurses and midwives per capita 9. The PHC centers' operating hours do not include evenings and nights.		
	O	1. Existence of migrants in Iran with degrees in medical sciences who can serve the migrant population. 2. Iran's telecommunications network growth and the presence of knowledge-based businesses developing health applications 3. Iran has reasonably priced transportation.		
	T	1. The availability of free services in health centers has led to an increase in Afghan migrants visiting PHC facilities. 2. Population aging and increased pressure on the health system 3. Economic crises might compromise the stability of the healthcare system.		

**TABLE 4:** Strengths, Weaknesses, Opportunities, and Threats and recommendation for the ability to pay and affordability [19] (Iran, 2023).

	SWOT		Recommendation based scoping review and interview	Details
Ability to pay and Affordability	S	1. Provided PHC services free of cost or a modest fee. 2. Full cover of the premium of Refugees with a specific disease and vulnerable refugees 3. Refugees' ability to get free rehabilitation services through CBR programs 4. Access to subsidized services in the public sector	• Modifying the qualifying requirements (W 3) ○ (a) Increasing the income threshold for entering health insurance ○ (b) Increasing the number of eligible demographic groups • Making the premium affordable (W 1) ○ (a) Subsidy (The government pays the premiums, indirect tax, and donation to premiums) ○ (b) Sliding scale of premiums (W 10) • Modifying enrolment (W 2, 3) ○ (a) Simplifying the enrolment procedure (W 8, 9) ○ **(**b) Integrating sources for enrolment (W 7) ○ (c) Changing the unit of enrolment ○ (d) Improving premium collection approaches • Other cost-cutting tactics include strategic and customized purchases of medications or medical devices from exclusive companies at unique and wholesale pricing. The pharmaceutical costs of communities are dominated by a segment of all utilized medicines. Such purchases for refugees can save service costs, and pharmaceutical companies welcome them because they have loyal consumers whose insurance companies will only reimburse their products. Pay for performance (P4P), the lowest comparator price to more expensive medicines. (W 5, 6) • Reimbursement of patient expenses: Partially or completely covering direct and indirect costs of primary healthcare access. • Management of the case: Individual patients are assigned a healthcare provider (e.g., a nurse or a social worker) who assesses their needs, assists in the creation of care plans, facilitates access to comprehensive services (including but not limited to primary healthcare), coordinates ongoing care, monitors patients, and advocates for them.	-Appendix A. Sections A.3, A.4, A.5.4 and related sub-sections and quotations. -Appendix B
	W	1. Unsustainable financial resources 2. Inappropriate Pooling of Monetary Resources and Health Risk 3. Problems related to designing administrative and organizational processes 4. Non-alignment between Afghan refugees’ burden of diseases and financial and insurance planning 5. Inefficient payment system 6. Inappropriate implementation of strategic purchasing policy 7. Inappropriate management of charitable and NGO resources 8. Enrolment prerequisites in the insurance plan 9. Insurance premium payments and other payments are made concurrently 10. Taking full payment of the insurance cost rather than making installment payments		
	O	1. There are multiple financing options to assist migrants' health. 2. There are many approaches that can be used to make the premium affordable		
	T	1. Inappropriate method for allocation of international aid and budgets resulting in Iran's insufficient share 2. Low economic growth 3. Harmful Effects of economic sanctions on the health system 4. High inflation in the country's economy and health sector 5. A significant proportion of the informal economy in Iran 6. The pattern of diseases in the Refugee families 7. A huge number of vulnerable migrants and inappropriate identification 8. The high cost of treating refugees and its adverse effects on the healthcare system		

### Approachability and Ability to Perceive of Migrants

Iran has approved most international conventions relating to migrants. The country is currently home to 3,400,000 refugees, and the majority of them are Afghans [4]. It is estimated approximately 2.1–6 million undocumented Afghans are living in Iran [23]. Since the recent upheaval in Afghanistan, the number of Afghans seeking international assistance has increased remarkably. Accordingly, it has been estimated that 500,000–1,000,000 have fled to Iran. By 31 December 2022, 5.2M million refugees and asylum-seekers from Afghanistan are reported in neighboring countries [24, 25]. These refugees need urgent help and support to meet basic needs such as food and shelter. Despite domestic constraints and economic challenges, Iran has been able to carry out many interventions emphasized by the conventions. However, UHC for migrants is still a long way off. Due to the lingual, cultural, social, and religious similarities between the Iranian and Afghan people, access to healthcare for Afghan migrants has not been a considerable challenge in Iran [26]. For instance, female patients can seek healthcare services through female providers. Moreover, there is no requirement for a legal certificate to receive PHC services, documented migrants have the right to use recognized healthcare facilities, and undocumented migrants are not exposed to any legal restrictions; however, other issues that have been mentioned prevent them from using the service.

One of the greatest obstacles to migrants’ access at this level has been the absence of a coherent assessment of the health status and health risks of recently arrived migrants, as well as a consistent program to inform and familiarize them with the health rights and facilities in Iran’s health system. It is highly uncommon to introduce migrants to and raise their awareness of Iran’s healthcare system through group visits, as opposed to giving them individualized care. In addition, while being one of the community-based initiatives that use volunteers to improve population health, the potential of health ambassadors have not been meaningfully utilized for migrants [27]. One subject that requires capacity building, especially for vulnerable groups, is weakness in the proactive identification of health needs, especially for undocumented migrants, and inter-organizational/inter-sectoral care pathways, notably between migration organizations and the health system.

Opportunities to expand access at this level include migrant health volunteers, migrant communities where residents are concentrated, and the integration of migrants within the society rather than residing in the camps and isolation. In addition, the concentration of migrants in specific provinces and neighborhoods, plus the wide internet and mass media coverage can foster migrants’ access to health services. However, migrants’ access to healthcare has been compromised due to their poor health status in their country of origin, differences in health attitudes, marginalization, insufficient health literacy, identity, and residency status, and difficulties with employment contracts.

### Ability to Reach and Availability and Accommodation & Ability to Engage and Appropriateness

Comprehensive health centers in urban and rural settings are the main part of the primary healthcare system (PHC) that provides healthcare services to both Iranian citizens and migrants (mainstream model). The simultaneous presence of male and female healthcare workers and the availability of nutrition and psychology counselors at PHCs are the strengths of Iran’s health system at the first level of access. Lack of initial comprehensive health assessment or “welcome visit” to new migrants; not using mixed models of care that contain additional models such as specialized focus services and gateway services, and low number of health workers *per capita* are the most important weaknesses at this level of access to health services for migrants.

### Ability to Pay and Affordability

The deployment of inappropriate procedures in collecting, pooling, and allocating insurance premiums, along with inadequate resources have restricted migrants’ access to healthcare services at this level. We advocate removing such barriers by adopting a sliding scale of premiums and simplifying the enrollment process for migrants.

## Discussion

Integrating SWOT analysis and Levesque’s conceptual framework with country-specific examples can provide policymakers with evidence-based guidance for developing the most effective policies for migrants. Our findings revealed that health officials’ emphasis on the six building blocks of a health system has led to difficulties with people’s access to health services, particularly for migrants [25]. Insufficient system thinking approach in policymaking for the health system has shown a negative impact on the health system’s outcome. We advocate policymakers consider both the supply (health system) and demand (people) sides when establishing programs and policies. There are sid-systems, sub-systems, and stakeholders on both the supply and demand sides that have an impact on the investigated system, both positively and negatively. A study on the System Dynamics Approach to Immunization in Uganda found that many subsystems influence the success of the immunization program, including mothers’ level of literacy, the effect of the media, the level of civil unrest, transportation constraints and availability and costs, and poor incentives for health workers [29, 30].

By scheduling a welcoming visit to basic healthcare facilities, it is feasible to evaluate the present health situation and health risk assessment for recently arrived migrants [31]. Among Afghan refugees, there are many volunteers available to provide services to migrants. Inter-organizational/inter-sectoral care pathways, group visits instead of individual care, community health workers, and document-issuance facilitation can all be used as interventions to enhance awareness of health plans and benefits. It will significantly reduce the barriers to access those migrants face as a result of the approachability [15]. In addition, simplifying financial processes for migrants, for example, innovative ways for revenue generation, paying the insurance premiums payment in installments, simplifying the enrollment procedure [32, 22], integrating enrollment sources, changing the enrollment unit, and improving premium collection approaches can help them to manage their spiraling expenses [33].

### Recommendation

Despite initiatives to ensure that everyone has access to healthcare, there are still inequalities between migrant and non-migrant populations in terms of their ability to obtain services [1]. Although fluctuating, during the last two decades, over 10% of people have been uninsured in Iran [28]. Worse still, 2 to 5 million migrants, most of whom are uninsured, and a significant portion of the informal economy have challenged obtaining adequate health coverage for migrants. We propose adopting mixed healthcare models, e.g., specialized focus services, gateway services, and restricted services, to overcome the high number of migrants, financial crises, migration cycles, places of migrants’ entry, and the concentration of migrants in certain provinces [29]. Such an approach might also help address various health needs of migrants in their first year of arrival and the following years [34].

Migrants may live and work in different countries until permanent settlement. We propose the UNHCR to pave the way for the establishment of international or regional multinational social insurance firms to deal with migrants’ health insurance and pension continuously and efficiently. This mechanism may reduce migrants’ low willingness to pay insurance premiums due to their uncertain plan to either move to another country or return to their homeland.

### Limitations

Migrants’ health is considered a complex and non-linear global health concern with many variables that could change over time. Our approach to SWOT analysis does not take into account the long-term outcomes and consequences of policies or interventions. Although it can provide beneficial insights into short-term changes in health outcomes, there is a limited capacity to predict the effects of changes on migrants in the long-term future. Accordingly, we advocate future studies to address the precise health status and needs of migrants, financial reevaluation, and redesign of regional and national insurance policies to create more effective insurance policies for better healthcare services coverage of migrants. We tried to be as diverse as possible in selecting the interviewees and tried our best to include Afghans with and without, insurance, as well as having and not having a chronic condition, etc. As over 80% of Afghan migrants in Iran are under 45 years old, we only included this range age in our study. We could not take into account various Afghan ethnic groups while selecting them for interviews.

### Conclusion

Iran has adopted the mainstream services model to provide essential healthcare services to migrants regardless of their legal status. Despite its advantages, this strategy has specific challenges in providing the migrant community with proper health coverage. To ensure greater sustainability and better health outcomes, in line with our findings, we propose a mixed-method strategy to enhance migrants’ equitable access to healthcare services. Further, capacity building, promoting migrants’ engagement in decision-making for health, improving health literacy, proactive identification of health needs, and facilitating the financial process are all important components of ensuring that migrants have access to the healthcare they need in Iran.
