# Supplementary material for: Health System Response to Refugees’ and Migrants’ Health in Iran: A Strengths, Weaknesses, Opportunities, and Threats Analysis and Policy Recommendations
Source: Int J Public Health. 2023 Sep 28;68:1606268. doi: 10.3389/ijph.2023.1606268 (PMC10568312; doi:10.3389/ijph.2023.1606268)
Supplement: Supplementary file 1 [file DataSheet2.docx]

**Appendix B**

**Providing Health Services to Refugees: a Scoping Review**

Scoping Review was conducted in five steps.

**Step one:**

Study questions include:

- What migration laws influence access to health services?
- How is healthcare services financing provided to migrants?
- What are the service delivery methods for migrants upon their arrival?
- What services are a priority for migrants?

We conducted a scoping review for better understanding of the subject in the global context, which enabled us to connect the policies in Iran with the worldwide policiesfor better understanding of the subject in the global context, which enabled us to connect the with the worldwide policies(1). The purpose of this scoping review is to assess the content and scope of existing evidence on the dimensions and challenges of providing health services to migrants.

**Step two:** Identification of relevant studies;

- Our research group comprised of a principal investigator (AT), one Co-I (AO), one main researcher and coordinator (AB, PhD), PhD researchers (AfT; MB), MD students (SS), and a Master's student in healthcare management (SK). We reviewed both documented and undocumented migrants. A scoping review of academic (databases) and grey literature (websites) in the English language was conducted by the research team. The literature search was organised around the following main principles, which included a time frame, databases, and search terms:
- **Study time frame**: We decided to run the study for the period of 2010 to 2021. This timeframe coincided with the new wave of migration, which was exacerbated by the decade-long wars and conflicts in Syria, Myanmar, Yemen, Afghanistan, South Sudan, and Venezuela, This helped us to understand the host countries' previous experiences in providing health services to new migrants; Afghanistan also experienced a more stable security and economic situation in the last decade compared to 2020 and 2021. This timeframe coincided with the new wave of migration, which was exacerbated by the decade-long wars and conflicts in Syria, Myanmar, Yemen, Afghanistan, South Sudan, and Venezuela, This helped us to understand the host countries' previous experiences in providing health services to new migrants; Afghanistan also experienced a more stable security and economic situation in the last decade compared to 2020 and 2021. The host countries' pre-2010 experiences have also been applied to the new wave of migration.
- **Databases and websites**: PubMed, Web of Sciences, Scopus, Embase, google scholar, ProQuest, UNHCR, IMO, WHO/EMRO/Europe, and any other available databases were included in the search. Also, the migration and health laws mentioned in the articles were followed up, and their text was analyzed.
- **The search terms**: TITLE-ABS-KEY : (refuge* or migration or immigrant* or foreign* or migrant* or asylum) AND TITLE-ABS-KEY: (healthcare or health-care or delivery or needs or coverage or (universal health coverage) or uhc or essential or services or service* or access* or planning or delivery) AND TITLE-ABS-KEY: (financ* or protection or fiscal or financing or Insurance or Insur* or cost or funding or fund).

**Step three:** SelectionSelection of included studies

We examined and considered articles on service delivery models, specific funding methods, financial, insurance, administrative, and migration laws. Studies of non-English languages and intra-country migration were overlooked. Systematic and comprehensive review studies in a specific dimension related to the health of migrants had a higher priority for selection.

**Step four and Step five:** Charting data; organizing, summarizing, and reporting results

To ensure the accuracy of data extraction, three researchers worked together. Two researchers extracted data and details, i.e., the study year, approach, first author, target population, type of health services, method of providing services, financial laws, migration laws, health service laws, and insurance laws. The third researcher re-checked the data extracted from the included articles to enhance accuracy.

- **B:Providing health services to migrants: A Scoping Review.**

1590 articles were found in the initial search 254 articles were duplicates and thus removed. The titles and abstracts of 1336 articles were reviewed, and 1155 articles were removed, being unrelated studies. Thirty-four studies were finally selected based on the study criteria. Fig 1 shows the process of searching and selecting studies. Selected studies were conducted between 2009 and 2022. Studies were conducted in Canada (2 studies), Australia (3 studies), United Kingdom (2 studies), Worldwide (4), Netherland (2 studies), Malesia (1 study), Thailand (1 study), USA (1 study), Czech Republic (1 study), Jordan (4 studies), Lebanon (1 study), Germany (3 studies), Europe (2 studies), Turkey (3 studies), Iran (3 studies) (Table 1).

**Full-text articles excluded, with reasons
(n =147)**

**Full-text articles assessed for eligibility
(n =181)**

**Records excluded
(n =1155)**

**Records screened
(n = 1336)**

**Records after duplicates removed
(n = 1336)**

**Additional records identified through other sources
(n = 32)**

**Records identified through database searching
(n = 1558)**

Identification

Screening

Eligibility

**Studies included (n =34)**

Included

**Fig 1. Flowchart for the systematic literature search**

**Table. 1 Main finding of selected articles**

| **Country / Reference No** | **Authors / Study approach /publication year** | **Target groups** | **Services covered or suggested** | | **Description of financial, legal, social, migration laws and models of health services delivery** |
| --- | --- | --- | --- | --- | --- |
| Australia  (2-4) | Diana Milosevic / professional/ 2012 | asylum seekers | Health needs commonly identified in refugees  and asylum seekers after arrival in Australia  include:   - psychological issues - nutritional deficiencies - infectious diseases - under-immunisation - poor dental and optical health - poorly managed chronic diseases - delayed growth and development in children - physical consequences of torture - gynaecological health needs | | - Most health assessments for newly arrived refugees occur at the primary care level, either in private general practice, through state-funded community health centers with salaried general practitioners or through dedicated refugee health services. - The NSW Refugee Health Service is one example of a specialised unit providing dedicated refugee health services. - Patient factors affecting access include lack of familiarity with how our health system operates, language barriers, mistrust or anxiety and financial constraints. - Provider factors may include time constraints, unfamiliarity with refugee health issues and inadequate interpreter use |
|  | Fair, G. L. /  Qualitative / 2018 | asylum seeker  (Refugee)  (In-country Special Humanitarian)  (Global Special Humanitarian)  (Emergency Rescue)  (Women at Risk) (Refugee Pending Bridging)  (Return Pending) visa (Temporary (Humanitarian Concern))  (Protection) visa. | The issues encountered by asylum seekers for healthcare | | - Asylum seekers had a difficult time transitioning to mainstream primary care. The complexity of health and immigration systems, the way asylum seeker–specific services provide care, mainstream general practitioner (GP) services' lack of understanding and accommodation, asylum seekers' own lack of understanding of the health system, mental illness, and social and financial pressures were all contributing factors. Conclusions: Asylum seekers need to be better prepared for the transition to mainstream primary care. To better accommodate the needs of asylum seeker patients, mainstream GPs and other clinicians need greater education and support. |
|  | Gillian Gould / Cross-sectional mixed-methods /2010 |  | Incomplete immunisations  HIV  TB  Hepatitis B & C  malaria  FBC  Schistosomiasis and Strongyloides serology  Nutrition  Iron/ vitamin D,A  Psychological history  effects of torture and other forms of trauma  Mental health problems  Hypertension  Pediatrics, child health care  breast and cervical screening.  Medical history  Examination: (a)Physical  (b) Cardiac, respiratory and abdominal  (c) Dentition: caries, gum disease, decreased dentition.  (d) Vision and hearing.  (e) Other: scars or injuries  The management plan includes: follow-up, initial recommendation, referrals to allied health professionals | | LAW   - health assessment is a voluntary one-off service and must be provided within twelve months of the person’s arrival in Australia, - Temporary visas are not covered by Medicaid and AIDS treatment.   FINANCIAL   - Medicare-funded, insurances - Fee: $208.70 Benefit: 100% = $208.70   PROVIDER   - The Australian Government provides free telephone interpreters for physicians who visit immigrants. It also includes the translation of medical records. - A multidisciplinary primary healthcare clinic used for newly arrived humanitarian entrants to provide health services. - The health assessment should be undertaken in a culturally sensitive manner |
| Canada (5, 6) | Michael Pysklywec / Practice/ 2011 | Migrant farm workers in Canada | Health issues in migrant farmworkers in Canada and the United States:  Musculoskeletal - Injuries - Pain in back, neck, knee, shoulders, hands or feet • Ocular - Conjunctivitis - Corneal foreign bodies and abrasions - Pterygia • Dermatologic - Contact dermatitis - Folliculitis - Tinea • Psychological - Depression - Anxiety - Inconsistent sleep patterns • Sexual and reproductive - Sexually transmitted infections (e.g., urethritis) - HIV infection. | | LAW:   - All documented migrant farmworkers have medical coverage in Canada because employers must provide such coverage as a condition of employment. - The type of nonoccupational coverage varies among provinces and program streams. - The IFHP provides federally funded healthcare coverage for refugees until they gain access to provincially funded health insurance.   FINANCIAL:   - They pay Canadian employment insurance premiums, pension plan premiums and income taxes.   PROVIDER:   - Health care needs are different for each group of immigrants, which can be considered in the design of insurance packages and plans. |
|  | H.P. Harris, debate/ 2015 | Refugee | The Canadian Health System has three levels for refugee health based on their source of sponsorship, country of origin, or claim status.  Tier 1: "Expanded Health Care Coverage"  Who is eligible:  • Government-assisted refugees (GARs) • Privately sponsored refugees (PSRs) who receive government funding (a minority of PSRs) • Victims of trafficking who have been issued a temporary resident permit • “Certain” individuals (on the Ministers initiative) who are being resettled in Canada on humanitarian grounds  Entitlements:  • Hospital services; services of licenced health care professionals; and laboratory, diagnostic and ambulance services (services covered until the individual becomes eligible for provincial health care) • Supplemental benefits—prescribed medications and pharmaceuticals, limited dental and vision care, prosthetics, and assisted mobility devices, home care, long-term care, psychological services, and post-arrival health assessments (benefits covered for as long as individuals receive federal government sponsorship)  Tier 2: “Health Care Coverage”  Who is eligible:• PSRs who do NOT receive government funding (majority of PSRs) • Individuals with accepted refugee claims • Individuals who have received a positive Pre-Removal Risk Assessment • Refugee claimants who are NOT from a designated country of origin (DCO)  Entitlements: Hospital services, services of licenced health care professionals, and laboratory, diagnostic, and ambulance services • Medications and vaccines ONLY if needed to prevent or treat a disease or condition that poses a risk to public health or safety  Tier 3: “Public Health and Public Safety Coverage”  Who is eligible:• Refugee claimants who are from a DCO • Individuals whose refugee claim has been rejected and who have exhausted their right to judicial review or appeals  Entitlements: Hospital services, services of licenced health care professionals, and laboratory, diagnostic, and ambulance services (ONLY if needed to prevent or treat a disease or condition that poses a risk to public health or safety) • Medications and vaccines ONLY if needed to prevent or treat a disease or condition that poses a risk to public health or safety. | | |
| All country (7) | Qingyue Meng/ systematic  Review / 2011 | Vulnerable groups were defined as children, the elderly, women, low-income individuals, rural populations, racial or ethnic minorities, immigrants, and population with disability or chronic diseases. | Expanding health insurance coverage in vulnerable groups | | - 1. Modifying the eligibility criteria 1a. The increasing income threshold for entering health insurance 1b. Expanding the categories of eligible population groups 2. Increasing awareness of schemes and benefits 2a. Awareness campaign by advertisements in media 2b. Awareness campaign targeted to specific places 3. Making the premium affordable 3a. Subsidy 3b. Sliding-scale premium 4. Modifying enrolment 4a. Simplifying enrolment procedure 4b. Integrating sources for enrolment 4c. Changing unit of enrolment 4d. Improving premium collection approaches 5. Improving health care delivery 5a. Improving health care package 5b. Controlling price of services 5c. Improving the quality of services 6. Improving the management and organization 6a. Improving information system 6b. Staff training 6c. Transparent management |
| England (8, 9) | Kor Grit / Comparative Policy Analysis / 2012  Rafighi / qualitative study design with  a social justice perspective / 2016 | - Undocumented Migrants - ‘vulnerable migrants’ (ie, defined as adult non-EEA asylum-seekers, refugees,   undocumented, low-skilled, and trafficked migrants susceptible to marginalized healthcare access) | - Access to Care:   1. Primary care (general practitioner services)  2. Nonemergency secondary care  3. Emergency secondary care  4. Accident and Emergency department  5. Communicable diseases  6. Mental health care   - Health Service Principles | | - Reimbursement Provider:   1. (in case of registration: NHS funding)  2. ….  3. ….  4. NHS funding  5. NHS funding except for HIV treatment  6. (in case of compulsory care: NHS funding)   - 1. All asylum-seekers and refugees are entitled to register with a General Practitioner and receive free NHS hospital treatment. 2. GP practices retain discretion to register refused asylum-seekers and provide them health services to the same extent that they have this discretion in registering and providing services to any patient, regardless of residency status. 3. Treatment of certain specified communicable diseases (eg, tuberculosis, hepatitis B, measles), compulsory mental health treatment, treatment provided in the event of an accident, and emergency department services are exempt from charges for all patients. 4. Health professionals must not discriminate against asylum-seekers or refused asylum-seekers by unfairly prioritizing other patients over them. 5. In England, refused asylum-seekers and asylum-seekers not receiving benefits may still be entitled to free prescriptions. Prescription charges have been abolished in Wales, Scotland, and Northern Ireland. 6. Different entitlements to free hospital treatment for refused asylum-seekers exist in each UK nation. 7. It is not the responsibility of doctors to make decisions concerning the eligibility of patients to access free NHS hospital care. 8. Refused asylum-seekers who were undergoing a course of hospital treatment at the time their claim for asylum was rejected are entitled to undergo that period of treatment free-of-charge until completion. |
| Netherla nds (8, 10) | Kor Grit / Comparative Policy Analysis / 2012  J. Suurmond / A qualitative study / 2013 | Undocumented Migrants / newly arrived asylum seekers | - Access to Care:   1. Primary care (general practitioner services)  2. Nonemergency secondary care  3. Emergency secondary care  4. Accident and Emergency department  5. Communicable diseases  6. Mental health care   - the specific issues that healthcare providers need to address in the first contact with newly arrived asylum seekers. | | - Reimbursement Provider:   1. Linkage Act fund  2. Dubious debtors (Special budget)  3. Dubious debtors  4. Dubious debtors  5. Dubious debtors (or Linkage fund)  6. (in case of compulsory care: government funding)   - Healthcare providers identified four issues they aimed to address in first contacts with asylum seekers: (1) assessing the current health condition; (2) health risk assessment; (3) providing information about the healthcare system of the host country; and (4) health education |
| USA (11) | Nancy Berlinger / 2013 | Undocumented Migrants | Access to Care  With special attention to Emergency secondary care | | Undocumented immigrants do not have access to public, supportive, or even private insurance. Most vital services are provided to these people in emergency centers under the EMTALA Act. In 1986, Congress enacted the Emergency Medical Treatment & Labor Act (EMTALA) to ensure public access to emergency services regardless of ability to pay. |
| world, particularly in  middle-income countries (12). | Paul Spiegel / Debate / 2018 | Refugee | - Innovative health financing for refugees | - If the health system of the host country does not have enough capacity, a parallel system can be used (should be avoided if possible). - Traditional funding for inadequate humanitarian emergencies is unsustainable and mainly provided by high-income countries (HICs). - How and what type of refugee health care is established depends upon the contexts - Risk-retention (refugee host countries are responsible for risk)   - Dependent upon planning: ● Domestic contingency funds or budget allocations: money for emergency relief set aside prior to event ● Taxes and subsidies to alter incentives for providing funding ● Line of contingent credit: a loan disbursed under certain circumstances   - Not dependent upon planning: ● Budget reallocation ● Tax increases ● Post-emergency credit ● User fees ● Taxes and subsidies to alter incentives for providing funding ● Tariffs or subsidies to alter prices of goods during emergencies - Risk transfer (refugee host countries transfer risk to another entity)   - Dependent upon planning: ● Traditional insurance or reinsurance: a contract where the insured pays insurer a premium, and the insurer agrees to pay for pre-specified and post-verified losses ● Indexed insurance: insurance contract where insurer makes payments based on certain external, measurable parameters or index ● Capital market instruments: financial instruments that can be bought or sold on capital markets, and investors shoulder risk (e.g., catastrophe bonds and swaps, Pandemic Emergency Financing Facility) ● Contingency pooled UN funds (e.g., Central Emergency Relief Fund and Country-Based Pooled Funds)   - Not dependent upon planning: ● Discretionary post-emergency aid: includes in-kind and cash transfers Discretionary post-emergency aid is the most common instrument for aid delivery in humanitarian emergencies and is provided primarily by HICs - International humanitarian assistance is paid annually while crises are multi-year - Improving health financing for refugees requires a paradigm shift towards pre-emergency and multi-year planning using risk-transfer instruments - There are various types of insurance schemes, from those that are publicly funded through some form of taxation (public insurance) to privately funded types (private insurance). Types of enrollment (mandatory, voluntary), contributions (income-based, community-based, risk-based), and management (public, non-profit, for-profit commercial, non-profit community) vary accordingly. - In general, refugees should be provided with a similar level of services to that of the “average” national In most countries where refugees are located, it is unlikely that the “average” national can afford private health insurance. - Microinsurance refers to public, private, not-for-profit, or community-based insurance schemes whose services operate at the local level and are specified to the needs of the poor. It targets those who would generally be excluded from mainstream insurance coverage. It protects the vulnerable from risks specific to their situation - Combined indexed insurance and catastrophe bonds are used to finance the health of migrants in emergencies such as natural disasters and epidemics. The maturity of the bonds can be several years. - Pay for performance is recommended for special services such as vaccination, safe delivery, malaria, etc. | |
| World (13, 14) | D. Gil-González / systematic review / 2015  Smithman / typology / 2020 | - Migrants - vulnerable populationsa | - Barriers to health care for migrants - Organizational innovation components to improve access to primary healthcare for vulnerable populations | - The lack of health insurance and the high cost of medicines (non-universal health system) were cited as structural impediments, as were organizational features of the health system (social insurance system and national health system). Individual barriers included linguistic and cultural differences. - The final typology included 18 organizational innovation components, the majority of which focused on Availability & Accommodation (7/18), Approachability (6/18), and Acceptability (3/18). Navigation and information, community health workers, one-stop shops, case management, group visits, cost-sharing, primary healthcare brokerage, and other elements were included. | |
| Czech Republic (15) | Dagmar Dzúrová / cross-sectional survey / 2014 | Immigrants | - Access to Health Insurance: | - Thirty percent of immigrants eligible for Czech, public health insurance due to permanent residency/asylum were not enrolled, and fifty percent of those eligible due to their job status were not enrolled. Migrants with a limited command of the Czech language are more likely to be excluded from the public healthcare system. Instead, people either stay in the commercial health insurance system or pay for both commercial and public health insurance at the same time, which is extremely inefficient. Since there are no compelling reasons to remain outside of the public health insurance system, a lack of understanding is determined to prevent eligible immigrants from enrolling. | |
| Jordan (16-19) | Margaret Ewen / 2014  Shannon Doocy / cross-sectional survey/ 2015  Ay, M / cross-sectional, analytical, observational /2016  Al-Rousan / mixed methods / 2018 | Refugees | - Medicine procurement prices - Access to NCDs care - Most needed health care services - Public health issues of the refugee | - Price assessments focused on the top 80 medicines by value, which accounted for 93 percent of the General Fund's pharmaceutical spending. UNRWA prices were 0.99, 1.00, 0.98, and 1.12, respectively, when compared to Management Sciences for Health's International Drug Price Indicator Guide, Jordan's Joint Procurement Department, Persian Gulf Cooperation Council, and IDA Foundation bulk packs. Applying the lowest comparator price to five drugs with higher prices would lead to a USD1.4 million savings. - In Jordan, 84.7 percent (CI: 81.6–87.3) of the 1363 NCD patients were treated; more than half of Syrian refugee households in Jordan had a member with an NCD. A considerable number of people did not undergo treatment, claiming cost as the main reason. - Chronic diseases and dental problems were common, in addition to the prevalent acute and communicable disorders. Advanced services were more difficult to obtain than preventive and primary health care. Access was limited by structural and financial barriers. - Syrian refugees ranked cost as the most significant impediment to receiving health care. More resources should be directed toward chronic diseases and mental health, according to both refugees and health care providers. | |
| Lebanon (20) | Hala Ghattas/ cross-sectional survey/ 2015 | Refugees | - food security | - About 41% (CI: 39-43) of households reported being food insecure, and 20% (CI: 18-22) were severely food insecure. | |
| Germany (21-23) | Maren Mylius / survey / 2015  Bauhoff, S. / Quantitative / 2018 | - Migrants - Asylum-seekers - Asylum-seekers | - Migrants with communicable diseases - utilizations and costs of care - examine Electronic Health Insurance Card (EHIC) impact on the use of medical services by asylum seekers | - Migrants who do not have a valid residence visa are de facto excluded from access to healthcare in Germany. In the event of sexually transmitted illnesses and tuberculosis, there is one exception in the relevant legislation. In the event of evident need, the lawmaker has directed local Public Health Authorities to provide free and anonymous counseling, testing, and, if necessary, treatment. Vaccinations that are recommended may also be provided free of charge. To protect the right to health care, national legislation should be simplified, especially for undocumented migrants. - Asylum seekers had a higher rate of hospital and emergency department admissions, including admissions that may have been averted with better outpatient care or prevention. Their average spending was 10% greater than that of the regularly insured, owing primarily to higher hospital costs; however, there was significant variance in spending by place of origin. Facilitating asylum-seekers access to health care, particularly outpatient and mental health treatment, could improve their health and integration, possibly at a reduced cost. - Asylum seekers with an EHIC are substantially more likely than those with healthcare vouchers to seek ambulant medical care. Conclusions: According to the findings of this study, having to apply for healthcare vouchers at a social security office may be a significant barrier for asylum seekers. | |
| Europe (Austria,  Greece, Ireland, the Netherlands and the UK) (24, 25) | C.A. O’Donnell / descriptive comparative analysis / 2016  Chiarenza / mixed method approach / 2019 | - Marginalised migrants - refugees and migrants in Europe | - primary care - Supporting access to healthcare | - Policy guidance on migrant health was available in all countries. RESTORE researchers conducting fieldwork with health care practitioners, on the other hand, found that practitioners "on the ground" had little knowledge of such guidelines.   - The ability of primary care to provide care to migrant populations is influenced in part by migrant populations' legal rights to health care. While asylum seekers and migrants with permission to be in the host country had a legal right to health treatment, the situation for unauthorized migrants was significantly different. Only the Netherlands and the United Kingdom provided primary care to undocumented migrants, and even then, only for "medically necessary" treatment.   - Requirements for migrants to make out-of-pocket payments, register with a GP and have a choice of GP mirrored that of the wider primary care system of the country. Thus, countries that make their indigenous population register with a GP (the Netherlands and the UK) applied this requirement to migrant populations as well. Countries with a reliance on private expenditure and out-of-pocket payments (Austria, Greece and Ireland) require some migrant groups to make such payments.   - The structural configuration of health systems impacts migrants’ access to health care, over and above the day-to-day routine configuration of services such as practice appointment systems or issues of communication within the consultation.   - Some studies found that doctors could be reluctant to accept lower fees for caring for migrants.   - Workforce development was another key feature that could inhibit or promote migrant use and access to primary care.   - Within the process dimension, out-of-pocket payments, which are generally required upfront, impact migrants’ ability to access primary care. Navigating health care systems can also be complex. Registration procedures, appointment systems and the need to negotiate access with reception staff all add to the burden of accessing care for migrants. - The various actors providing healthcare to refugees and migrants confronted challenges related to the three stages of migration: arrival, transit, and destination. Regulatory, financial, and administrative barriers affected access to healthcare services; a lack of interpretation and cultural mediation services; a lack of reliable information on migrant patients' illnesses and health histories; a lack of knowledge of entitlements and available services; and a lack of organization and coordination between services. Access to specific services, such as mental health, sexual and reproductive care, child and adolescent care, and victim of violence care, was severely hampered by these restrictions.. | |
| Turkey (26-28) | Ekmekci, P. E. / debate / 2017  Torun, P. / mixed methods approach / 2018  Assi, R. / qualitative study / 2019 | Syrian Refugees | - Crucial effects of Turkish health and migration laws on Syrian refugees’ access to public health services - Health and health care access for Syrian refugees living in İstanbul - Health needs and access to health care: the case of Syrian refugees in Turkey | - The Turkish Constitution recognizes health as a human right. The refugee legislation are in many ways UN and EU compliant. So the 1951 Geneva Convention and 1967 Protocol social rights apply to refugees.   The Turkish Ministry of Health issued a ‘‘Circular on Health Services for Temporary Protection" to ensure Syrian immigrants' access to healthcare. But these laws lack clarity and are in conflict with international law.  The camps are in ten Turkish border towns. In 2014, these cities had 42,241 tents and other shelters in 21 camps. Primary care is provided by CHCs, whereas secondary care is provided by field hospitals and polyclinics. Primary health care includes routine child immunizations, child and maternal monitoring, reproductive health treatments, and health counseling. Syrian refugees living in camps have free access to primary and secondary health care. If more extensive care is required, patients are sent to public hospitals for free. Outside the camps, refugees have free access to primary and secondary health care.  The tremendous increase in territory population has put a strain on health care, both in terms of human and logistical resources. In 2015 alone, around 500,000 Syrians were moved from camps to public hospitals. 35,000 Syrians gave birth in Turkey, says the Ministry of Health. Syrian refugees occupied 30–40% of hospital beds in Syrian border provinces. Aside from hospital overcrowding, locals worry about Syrians devouring health resources and preventing ‘‘true" right holders, Turkish people, from receiving required services.  Reproductive services require special care.  Pregnant and postpartum refugee women get free iron and vitamin D supplements. Community health centers also provide free reproductive health counseling and contraception products. The UN Population Fund (UNFPA) has created Arabic leaflets on safe motherhood, pre and post-partum care, healthy eating throughout pregnancy, and safe sex attitudes. Syrian refugee newborns are also screened at community health facilities. 40.000 neonates will be tested for PKU, hypothyroidism, and congenital deafness in 2014. Newborns receive micronutrient supplements, including vitamin D and iron. These services are free at refugee and community health centers.  Syrian refugee health care in 2011–2015 : 7,519,668 outpatients 299 240 inpatients 48,193 births 226 513 operations 353,041 polio immunizations (0–15 years)  Leishmania and measles are frequent among Syrian refugees in Turkey. Other infectious diseases affecting refugees include hepatitis A, TB, malaria, and smallpox. In 2013, the city with the most Syrian refugees, Gaziantep, had the most measles cases. In 2015, 215 Syrian refugees were diagnosed with TB. From 2011 until date, 597 Syrian refugees have TB. However, the numbers are unreliable and invalid because they are primarily taken from refugee camps, where only 15% of Syrian refugees reside. The remaining 85% of Syrian refugees are spread among Turkish cities. Not all refugees are represented in data, but only those in camps.  In 2014, a widespread oral polio immunization programme was initiated. In 7 Syrian border cities and 5 cities with refugee camps, all children under 5 years old received 2 doses of oral polio vaccine. Regardless of age, all Syrian border crossers receive one dose of oral polio vaccine. Syrian refugees of all ages are also vaccinated against measles, mumps, rubella, tetanus, and diphtheria.  Most Syrian refugees work unregistered in agriculture, industry, and small businesses. These are part-time occupations with no benefits. The laws governing the hiring of refugees in temporary protection are inadequate. The enormous influx of desperate workers reduces job chances for both locals and refugees.  The Syrian migrants' official registration is vital for access to services, particularly health. Starting with the first Syrian refugees, camps have been meticulously registered. But it's hard to say the same for those who avoided camps. Deficient national data systems have led to repeated registrations by government agencies. Moreover, some refugees are afraid of being deported if they are properly registered. Others don't register because they don't know how. This registration disarray has left the number of Syrian refugees in Turkey unclear. With GDMM, an online registration system for all refugees is created. Syrian refugees must register online to obtain public services, including health. This system improved Syrian refugee registration and supplied more data.   - • The biggest issues were rising rent and a language barrier. Almost half of the women interviewed (49.6%) were unaware of Syrians' right to free healthcare. In the 30 days prior to the interview, 58.6% of participants visited state hospitals, primary health care centers, and pharmacies. This was hampered by a lack of knowledge of the Turkish healthcare system and a lack of language proficiency among the participants hospital wait times and staff negativity affected satisfaction. Most Syrian women (80.2%) were registered with the authorities, allowing them to use services. Assuring the other household members' registration status was not expected. Almost half of the women (49.6%) were unaware of the right to free health care. Those in the know got their information through friends, neighbors, or relatives (57.8 percent ). Among the 85 women polled, 24 (28.2%) said they utilized social media to learn about their service rights. - • The Turkish government has implemented much legislation for Syrian refugees, allowing them free access to emergency care units and primary, secondary, and tertiary healthcare centers in all 81 Turkish provinces. Language problems, refugee mobility, and legal restrictions hinder the effectiveness of refugee healthcare services. Mental health and rehabilitation services are lacking due to a lack of skilled professionals. | |
| I.R.IRAN (29-31) | Mohammadi/qualitative study / 2017  Azizi, N. / qualitative / 2019  Takbiri / exploratory / 2020  Kiani / mixed-method / 2020 | - Afghan women surviving near-miss morbidity - Refugees - Afghan immigrants | - Insufficient medical attention - experience of Afghan refugees in Iran regarding PHC delivery - The challenges of providing primary health care - Refugees and Sustainable Health Development | - From April 2013 to May 2014, a qualitative study was undertaken at university hospitals in Tehran. When women recovered from near-miss morbidity that happened around the time of childbirth, 11 Afghan women and 4 husbands were questioned. The WHO maternal near-miss technique was used to identify mothers prospectively. Mistreatment in the form of discrimination and inadequate medical care were two of the most traumatic events. Despite repeated requests for care, participants frequently reported poor women–professional communication and delays in recognizing maternal problems. Financial restrictions, high-cost care, a lack of health insurance, and inadequate literacy were all experienced as minor barriers to care. The non-somatic repercussions of near-miss morbidity had a long-term impact on women and families. Near-miss survivors' accounts provided a fascinating insight into Afghan women's prenatal care in Iran. The challenge for the healthcare system and professionals is to offer equal and dignified care to ethnic minorities while also improving communication skills and attitudes toward them. Antenatal consultations are the most effective and appropriate way to combat Afghan women's health illiteracy. - There were four main categories and 12 subcategories identified, including (1) challenges prior to PHC delivery: a large number of children, high service cost, lack of medical insurance, access to health centers, appointment to get services, to breastfeed and pregnancy at the same time; (2) challenges during PHC delivery: understanding Iranian words, health care provider's behavior, delay in getting service in PHC centers; and (3) challenges after PHC delivery: referral patient, high referral patient, high referral patient. The findings revealed that Afghan refugees face numerous hurdles at every stage of the PHC delivery process. Awareness of such issues can aid medical workers in improving service delivery to Afghan refugees, as well as employing trained Afghani nurses to assist them. - The most common problems in providing PHC to Afghan immigrants were broken down into three categories: individual, organizational, and society. At the individual level, communication obstacles and socioeconomic characteristics arose. The lack of insurance coverage for all immigrants and the lack of a screening procedure when immigrants arrived from the border were the key organizational problems. On a societal level, negative attitudes toward Afghan immigrants were a significant concern. - The health of refugees in Iran is supported by international and upstream policies, regulations, and practical projects. The majority of healthcare services given in Iran's PHC network are free to refugees and immigrants. They can also receive curative and rehabilitation therapies, the cost of which is determined by their level of health insurance. The government authorized all registered refugees to join the Universal Public Health Insurance (UPHI) scheme in 2015. Furthermore, 786 (389.7) impaired refugees used CBR services on average. 112,000 (30404.9) refugees were insured by the UPHI scheme on average. | |
| Thailand (32) | Tangcharoe / Lessons from the field/ 2017 | - Undocumented migrant workers | - health insurance | - • Problem Undocumented migrant workers are often ineligible for state social security programs; thus they must either postpone or pay for health care on their own. Approach, The Thai Ministry of Public Health, implemented a migrant health policy in 2001. Migrant health insurance is a self-funded program supported by an annual premium paid by workers. It allows undocumented migrants and their dependents to receive health treatment at public facilities and decreases catastrophic health costs. In the community and at work, a variety of migrant-friendly services, including trained community health volunteers, were implemented. In 2014, the government implemented a multi-sectoral migrant policy that was coordinated by the ministries of the interior, labor, public health, and immigration. The situation in the neighborhood Thailand's social security plan covered roughly 0.3 million employees in 2011 or less than 9% of the estimated migrant labor force of 3.5 million. Changes that are relevant According to the most recent data, 1 146 979 persons (33.7 percent of the total estimated migrant laborers of 3 400 787) applied, were inspected, and enrolled in the migrant health insurance plan between April and July 2016. Local communities value health volunteers recruited from migrant communities and workplaces, and they are beneficial in improving health and increasing migrant uptake of health services. The lessons I've learned Expanded health insurance coverage for undocumented migrants was made possible by the health ministry's ability to develop and administer migrant health insurance. Continued policy support will be required to enhance insurance enrollment and expand migrant-friendly services. | |
| Malaysia (33) | Chuah / qualitative / 2018 | - refugees and asylum-seekers | - access barriers   among refugees and asylum-seekers | - • Poor health literacy and lack of understanding of one's entitlement to healthcare, as well as language and cultural disparities, protection difficulties stemming from a lack of legal status, and inability to finance healthcare owing to inadequate livelihoods, are all major barriers to healthcare access. Overall, limited access to healthcare is thought to have a negative impact on the health of refugees, asylum seekers, and the host community and may result in higher healthcare expenses in the long run. | |

The following topics present the findings of the articles included in this study, which were the essential factors to be used in building a comprehensive model for providing health services to migrants.

- **B.1 Barriers to Healthcare Access;**
- **B.2 Appropriate health systems and services provider for migrants;**
- **B.3 Migrant Health Care Financing;**
- **B.4 Method of purchasing;**
- **B.5 Classification of migrants;**
- **B.6 Expanding coverage strategies;**
- **B.7 Components of organizational innovation to increase vulnerable populations' access to primary healthcare.**

**B.1 Barriers to Healthcare Access**

Migrants, healthcare professionals, policymakers, and other stakeholders all face different barriers in providing and receiving healthcare services. In addition, a migrant will confront a range of barriers at various points of his or her trip (before the travel, during the travel, the first year of residence in the destination country, and the following years of residence). The type of migration, such as documented and undocumented movement, migrant workers, academic migration, forced migration, war and conflict, temporary and permanent migration, and climatic migration, all influence the typology of barriers to health care access.

Barriers obstruct the process of receiving health care in a variety of ways. The Levesque conceptual framework for healthcare access provides policymakers with a clear perspective of the effect areas by including characteristics such as perception of health needs and desire to care, healthcare seeking, healthcare reaching, and healthcare utilization (Fig 2).


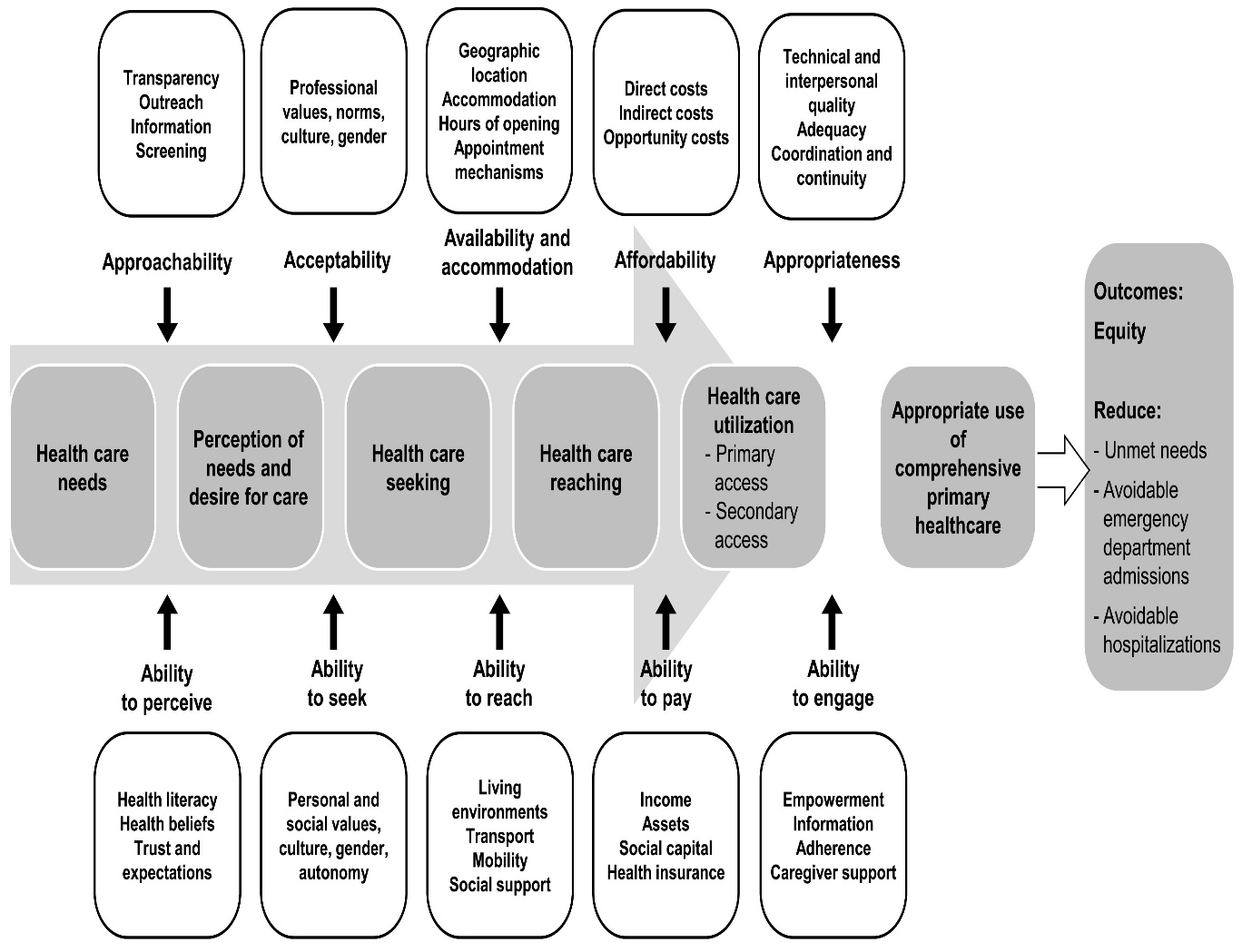


**Fig 2. The Levesque’s conceptual framework for healthcare access (14)**

The most important barrier for migrants to receive health services was their ability to pay, according to the articles included in the study and many others (13, 14, 18, 33). The host country's labor rules, migrants' insurance coverage, migrants' income and type of employment, tax legislation, and many other characteristics all affect migrants' ability to pay.

Other barriers that limit the ability to seek, receive, and communicate successfully between physician and patient, according to studies, are linguistic and cultural barriers. To tackle this problem, countries use free translators. Other barriers to migrants' access to health treatments include a lack of familiarity with the host country's health system and knowledge about accessible facilities.

Migrants' access to health services is influenced by various barriers and factors, including health system advancement and sustainability, immigration policies, health system productivity and performance, economic crises, financial assistance, level of education, migrants' health literacy, trained health workforce, and many others. Fear of identification and a lack of legal authority to access health plans were the main barriers for undocumented refugees, in addition to financial barriers.

**B.2 Appropriate Health Systems and Services Providers for Migrants**

The ultimate goal of providing healthcare to refugees is integrating them into the host country's national health system. If the host country's capacity is insufficient, parallel systems may be employed (should be avoided if possible). Housing status (temporary camps or integration with the host community; urban or rural) and the effectiveness of the district health system, as well as migratory stages, all influence the suitable health care systems for migrants (12), including:

- Preparedness (pre-emergency);
- Acute emergency
- Protracted (> 5 years)
- Durable solutions

o Voluntary repatriation

o Local integration

o Resettlement

When a large number of migrants arrive in a country for reasons such as war and conflict in their home country, the host country's health system has special priorities and measures in places, such as vaccination, communicable disease care and monitoring, injury treatment, and care for vulnerable migrants, such as pregnant women and children. As well as their needs for temporary housing. These first steps are being made to ensure the migrants' health as well as the health of the host country's community. However, as the second year of residence approaches, the health system faces new challenges, such as the interaction between country migration laws and healthcare delivery, documented and undocumented migrants, migrant health financing and insurance coverage, chronic diseases, migrant economic and social status, cultural differences, and migrants' communication and health literacy, all of which pose serious challenges to refugee-hosting countries.

There are four issues for newly-arrived migrants that health service providers should consider (10):

- Assessing the current health condition.
- Health risk assessment.
- Providing information about the healthcare system of the host country and
- Health education (15).

Employing specialized units to providing dedicated refugee health services is a strategy used by health systems to deliver health services in the early years of migrant arrival. These units are the channel for migrants to enter the health system of the host countries, and the existence of these centers has the following advantages:

- Their human resources for health usually receive fixed salaries
- Volunteer human resources are used in them in a coherent manner
- Their healthcare workforce is familiar with the culture and health problems of migrants
- Foreign nationals experience fewer language, cultural, and service delivery problems
- Foreign nationals in these centers get familiarized with the health system of the host country
- These centers train migrants to enter the host country's public health system.

The type of employment of refugees will affect the design and financial management of their health system. Refugees with specialized jobs will naturally receive more support through insurance organizations than refugees engaged in manual and temporary employment. Construction work and retail are common occupations among non-skilled refugees (34).

**B.3 Migrants’ Health Care Financing**

With the COVID-19 crisis, health systems and insurance companies have faced unprecedented challenges (35). Their overall accountability has diminished; also, existing financing for short-term humanitarian crises is unsustainable, and high-income countries are primarily responsible for providing it. The UN and international non-governmental organizations (NGOs) supply emergency foreign aid are the most common finance mechanisms currently in operation. Moreover, the conflicts have become more protracted (12).

**Refugees’ financing instruments can be expressed according to risk and time**

Risk and timing are the two most important aspects of the financing options available. Risk is defined as the possibility or likelihood of a loss, which can be associated with people or events. Risk-retention tools hold refugee-hosting governments accountable. They allow for more flexibility in payments because they can be spent whenever they want. Contingency funds, budget allocations, contingent credit, budget reallocations, increased taxes, and post-emergency credit are some of the instruments available. Risk-transfer instruments enable host countries to transfer risk to a third party. Because another entity is accountable for the danger, it gives better security. Insurance, reinsurance, bonds, swaps, and donations are some of the mechanisms that can be used. The other important factor is timing, which refers to when the harmful event occurs. Reserves, contingency funds, budget contingencies, contingent debt facilities, and risk-transfer products are examples of pre-emergency (ex-ante) instruments that rely on emergency preparation. Donations, budget reallocation, loans, and tax increases are examples of ex-post (after-emergency) tools that do not require emergency planning. A range of finance mechanisms are available for planning and responding to humanitarian emergencies, each with its own set of time and risk characteristics.

- **Risk-retention (refugees’ host countries are responsible for risk)**
  - *Dependent upon planning:*
    - Domestic contingency funds or budget allocations: money for emergency relief set aside prior to the event;
    - Taxes and subsidies to alter incentives for providing funding;
    - Line of contingent credit: a loan disbursed under certain circumstances.
  - *Not dependent upon planning:*
    - Budget reallocation;
    - Tax increases;
    - Post-emergency credit;
    - User fees;
    - Taxes and subsidies to alter incentives for providing funding;
    - Tariffs or subsidies to alter prices of goods during emergencies.
- **Risk transfer (refugee host countries transfer risk to another entity)**
  - *Dependent upon planning:*
    - Traditional insurance or reinsurance is a contract in which the insured pays a premium to the insurer in exchange for the insurer agreeing to pay for pre-specified and post-verified losses. There are many different types of insurance plans, ranging from government-sponsored (public insurance) to privately funded (private insurance). Enrollment (mandatory, voluntary), contributions (income-based, community-based, risk-based), and administration (public, non-profit, for-profit commercial, non-profit community) differ according to these factors. The major goal of refugee health insurance should be to integrate refugees into existing national healthcare systems, if they exist and are functioning. Traditional health insurance for refugees should be considered in a long-term setting when the health status is generally stable. In every society, there will always be vulnerable groups that cannot afford health insurance. It will be necessary to make decisions on who is vulnerable and who will help to pay (in full or in part) for these people. Depending on the number of refugees who contribute to the national health system, the risk pool may have become large enough to allow for the subsidization of insurance premiums and co-payments for these refugees, similar to what happens with nationals. UNHCR, which is now supporting millions of dollars in healthcare services through governments, NGOs, and faith-based organizations, which often provide parallel services.
      - Although the words "microinsurance" and "community-based health insurance" (CBHI) are sometimes exchangeable, microinsurance is a larger concept that encompasses CBHI schemes. Microinsurance refers to public, private, non-profit, or community-based insurance systems whose services are tailored to the requirements of the poor on a local level. It is aimed at those who are typically left out of regular insurance coverage. It protects the vulnerable from risks unique to their situation (e.g., flooding, catastrophic medical costs) depending on the likelihood and cost of the risk. Individuals pay minimum premiums to a small pool, which provides restricted coverage and a small but considerable payout. Microinsurance plans are frequently incorporated into existing social protection systems.
    - Indexed insurance: insurance contract where the insurer makes payments based on certain external, measurable parameters or indexes,
      - The indexed insurance window; Insurance funded by the private sector, multilateral and bilateral organizations, and UN agencies with explicit parametric indices might all be included in the indexed insurance window. The UNHCR, for example, spends millions of dollars each year on refugee health care. Some of these monies might be set aside for pre-emergency health insurance for host countries. The parametric indices must be produced and confirmed, but some examples include: 1) the Fragile States Index, which is a significant tool for identifying important elements that push a state into failure, and 2) a specific number of refugees crossing a border. However, extensive investigation is required to determine which indications, if any, are measurable and predictable. Funds should be sent to government-level offices that control health systems and are in charge of integrating refugees whenever possible. Existing health systems, whether fully functional or partially functional, will almost certainly require expanded capacity and receive support from the UN and NGOs.
    - Capital market instruments: financial instruments that can be bought or sold on capital markets, and investors shoulder risk (e.g., catastrophe bonds and swaps, Pandemic Emergency Financing Facility),
      - Bonds are a popular capital market mechanism in which a creditor lends money to a government, corporation, or other entity, which then issues a bond to the creditor. The bond is valid until a specific date (maturity date), after which the borrowed money (bond principal) are returned. Interest is normally paid out on a regular basis until the loan matures. The interest rate on bonds is either fixed or variable (coupon). A public entity, insurance business, or other institution issues catastrophe bonds to an investor. They usually have a high coupon rate because they are reinsuring another party. The investor defers or forfeits payment of the interest and/or principal if a disaster happens (today, the majority of these bonds are for natural disasters). Instead, the funds will be used to deal with the disaster. If no disaster occurs, the bonds normally expire after three years, with investors receiving their principal plus interest.
    - Contingency pooled UN funds (e.g., Central Emergency Relief Fund and Country-Based Pooled Funds).
  - *Not dependent upon planning:*
    - Discretionary post-emergency aid: includes in-kind and cash transfers discretionary post-emergency aid is the most common instrument for aid delivery in humanitarian emergencies and is provided primarily by HICs.

**B.4 Methods of Purchasing**

Other cost-cutting tactics include strategic and customized purchases of medications or medical devices from exclusive companies at unique and wholesale pricing. The pharmaceutical costs of communities are dominated by a segment of all utilized medicines. Such purchases for refugees can save service costs, and pharmaceutical companies welcome them because they have loyal consumers whose insurance companies will only reimburse their products (16).

Pay for performance (P4P) is a phrase that refers to funding efforts that attempt to improve the quality, efficiency, effectiveness, and total value of health services. It transfers financial risk from a traditional funder, typically the government, to a new investor who gives upfront funds to grow an evidence-based program for better outcomes. Service providers are given goals to meet. Achieving these goals will enhance service delivery while also lowering costs, with the savings being used by the local government or a donor to reimburse investment over time. In theory, investors take the risk since repayment comes only if the program is successful. Outcomes are measured using pre-defined parameters and confirmed by a third-party organization. Finance agreements are included in the P4P contracts, giving upfront capital to support service delivery throughout the project.

One study found that purchasing strategies can reduce costs. The top 80 drugs by value accounted for 93 percent of General Fund pharmaceutical expenditures. United Nations Relief and Works Agency (UNRWA) costs were 0.99, 1.00, 0.98, and 1.12 compared to the International Drug Price Indicator Guide, Jordan's Joint Procurement Department, the Persian Gulf Cooperation Council, and the IDA Foundation bulk packs. Applying the lowest comparator price to five more expensive medicines saves USD 1.4 million.

**B.5 Classification of Migrants**

One of the key factors of migrants' access to health care in all nations is the kind of visa and temporary and permanent residency status. Certain nations have imposed additional limits to maintain the long-term viability of insurance financing and combat the flood of temporary migrants seeking free health care. Representatives from the Ministry of Health, Welfare, and Sport (HWS) stated that non-urgent care for undocumented migrants should not be provided immediately because the Ministry of HWS believes that this will serve as an incentive for "health tourists" (people who travel from abroad to receive free medical care and then return home) (Netherlands) (8).

Resource limitations prevent countries from offering all health services to all migrants and classifying them based on migrant characteristics and sorts of access services, even in high-income countries. An example of this classification can be found in Canada. Refugee health in Canada is divided into three categories based on the source of sponsorship, country of origin, or claim status.

***"Expanded Health Care Coverage" is the first tier.***

**Who qualifies:**

- Government-assisted refugees (GARs);
- Government-funded privately sponsored refugees (PSRs) (a minority of PSRs);
- "Certain" persons (on the Minister's initiative) who are being relocated in Canada on humanitarian grounds;
- Victims of human trafficking who have been awarded a temporary residence permit.

**Entitlements:**

- Hospital services licensed health care professionals' services, as well as laboratory, diagnostic, and ambulance services (services covered until an individual becomes eligible for provincial health care);
- Additional benefits include prescription drugs and pharmaceuticals, limited dental and vision care, prostheses and assisted mobility devices, home care, long-term care, psychiatric services, and post-arrival health exams (benefits covered for as long as individuals receive federal government sponsorship).

***"Health Care Coverage" is the second tier.***

**Who qualifies:**

- PSRs who are not funded by the government (majority of PSRs);
- Those who have been granted refugee status;
- Refugee claimants who are not from a specified country of origin;
- Individuals who have obtained a positive Pre-Removal Risk Assessment (DCO);
- Hospital services, licensed health care professionals' services, and laboratory, diagnostic, and ambulance services are all covered;
- Medications and vaccinations are only given if required to prevent or treat an illness or condition that is harmful to the public's health or safety.

***"Coverage of Public Health and Public Safety" is the third tire.***

**Who qualifies:**

- - Individuals whose refugee claim has been denied and who have exhausted their right to judicial review or appeals;
  - Hospital services, licensed health care professionals' services, and laboratory, diagnostic, and ambulance services (Only if needed to prevent or treat a disease or condition that poses a public health or safety risk);
  - Medications and vaccines (Only if needed to prevent or treat a disease or condition that poses a public health or safety risk);
  - Advanced services were more difficult to obtain than preventive and primary health care access was limited by structural and financial barriers.

**B.6 Expanding Coverage Strategies**

One of the articles included in the study was related to strategies to expand insurance coverage for vulnerable groups (7), including migrants, which divided the experiences of countries around the world into six categories:

1. Changing eligibility criteria of health insurance;
2. Increasing public awareness;
3. Making the premium more affordable;
4. Innovative enrolment strategies;
5. Improving health care delivery;
6. Improving management and organization of the insurance schemes.

Each has a subset of different actions and strategies that allow policymakers to make choices tailored to the current situation. In countries expanding coverage, the categories identified from the literature can help policymakers consider their options, implement strategies where it is common sense, and establish appropriate implementation monitoring. These strategies are briefly mentioned in Figure 3 (7).

**
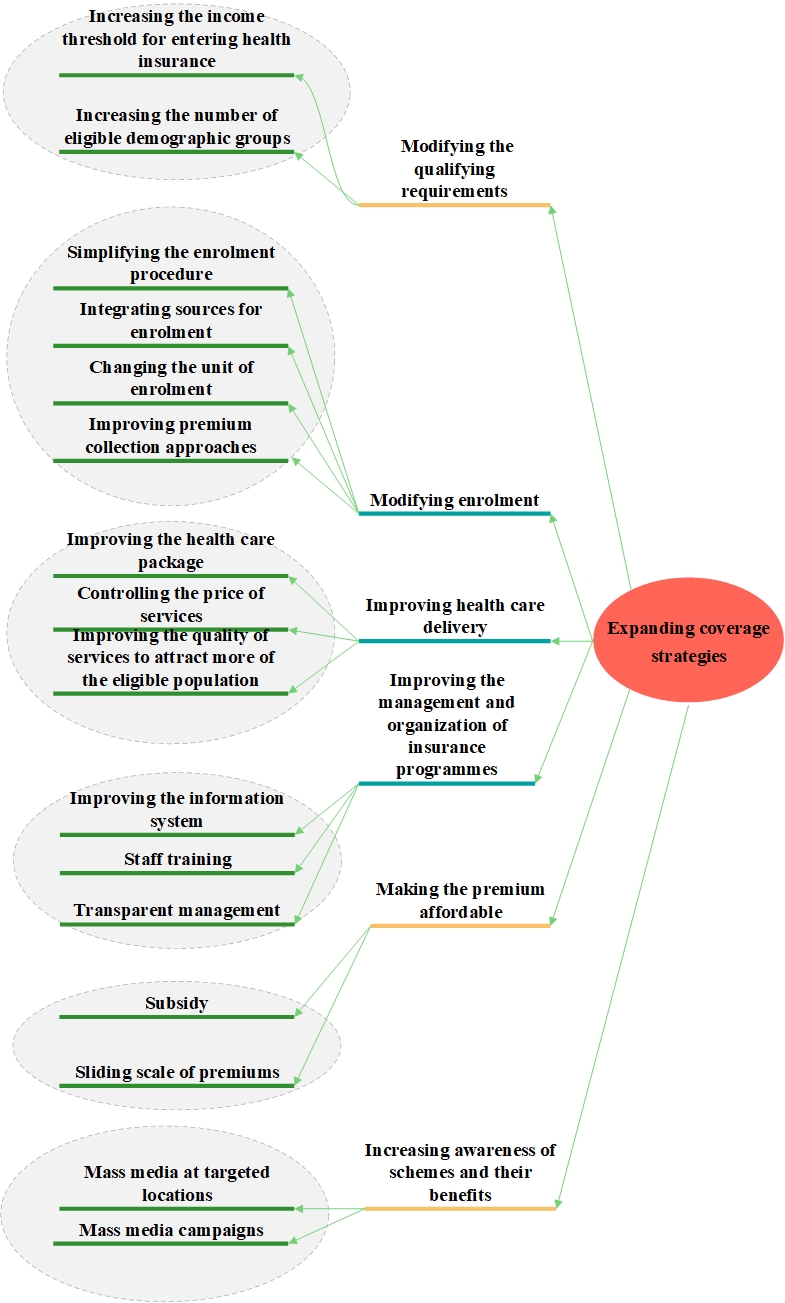
**

**Fig 3. The expanding coverage strategies^^[[1]](#footnote-2)^^**

**B.7 Components of organizational innovation to increase vulnerable populations' access to primary healthcare**

Various organizational innovations have been proposed to improve access to primary healthcare for vulnerable populations (14). It is separated into six categories, according to studies: Approachability, acceptability, availability & accommodation, affordability, appropriateness.

**Approachability**

1. Proactive identification of need: A mechanism is put in place to proactively identify vulnerable patients’ needs for primary healthcare and provide additional support to avoid the negative consequences of unmet needs.

2. Information and navigation: A service that informs and supports individuals on where, when, and how to obtain primary healthcare.

3. Brokerage of primary healthcare services: A service that assists vulnerable patients in connecting with a primary care provider or primary healthcare service, including single entry points to access and priority queueing based on vulnerability indicators.

4. Provision of primary healthcare services to the general public to reach vulnerable populations; primary healthcare services should be extended beyond the physical limits of primary care settings.

5. Inter-organizational/inter-sectoral care pathways: Primary healthcare organizations collaborate with other organizations (both within and outside the health system) to develop procedures that ensure vulnerable groups have timely access to needed services.

6. Making proactive appointments and maintaining proactive contact: Appointment-making processes in primary care that draw vulnerable patients in for care and keep in touch with them.

**Acceptability**

7. Services that are culturally appropriate by addressing linguistic or cultural barriers, primary healthcare can be tailored to the needs of a specific vulnerable group.

8. health worker in the community: A layperson, who is a trusted member of the community or has a thorough understanding of it, serves as a frontline worker who helps community members overcome cultural and linguistic barriers and gain access to primary healthcare.

9. Group visits Rather than providing individual care, primary healthcare is provided to a group of people with similar vulnerabilities or conditions.

**Availability & Accommodation**

10. Longer working hours to meet the needs of vulnerable populations: A primary healthcare organization extends its operating hours beyond 9 a.m. to 5 p.m.

11. Access to advanced features: A scheduling system that provides urgent care by a known primary healthcare team, triggers planned appointments when necessary and allows patients to schedule an appointment at the most convenient time.

12. Health-related virtual services: For consultations or monitoring health conditions, use videoconferencing, phone, email, text message, apps, and so on.

13. Services that are available on-demand: Patients who come in without an appointment can receive services.

14. Transportation services: Organizing transportation for patients who have difficulty getting to primary care facilities.

15. Task shifting or role expansion Upskilling: A healthcare worker who works with vulnerable patients on a regular basis to improve workforce capabilities. It's possible that formal providers' scope of practice will be expanded or that laypeople will be trained.

16. All-in-one solution: At the point of contact, multiple health and social services are provided in one location to provide comprehensive care to hard-to-reach vulnerable patients with complex needs.

**Affordability**

17. Reimbursement of patient expenses: Partially or completely covering direct and indirect costs of primary healthcare access.

**Appropriateness**

18. Management of the case: Individual patients are assigned a healthcare provider (e.g., a nurse or a social worker) who assesses their needs, assists in the creation of care plans, facilitates access to comprehensive services (including but not limited to primary healthcare), coordinates ongoing care, monitors patients, and advocates for them.

1. Peters MD, Godfrey CM, Khalil H, McInerney P, Parker D, Soares CB. Guidance for conducting systematic scoping reviews. Int J Evid Based Healthc. 2015;13(3):141-6.

2. Gould G, Viney K, Greenwood M, Kramer J, Corben P. A multidisciplinary primary healthcare clinic for newly arrived humanitarian entrants in regional NSW: model of service delivery and summary of preliminary findings. Aust N Z J Public Health. 2010;34(3):326-9.

3. Milosevic D, Cheng IH, Smith MM. The NSW Refugee Health Service - improving refugee access to primary care. Aust Fam Physician. 2012;41(3):147-9.

4. Fair GL, Harris MF, Smith MM. Transition from an asylum seeker-specific health service to mainstream primary care for community-based asylum seekers: a qualitative interview study. Public Health Res Pract. 2018;28(1).

5. Pysklywec M, McLaughlin J, Tew M, Haines T. Doctors within borders: meeting the health care needs of migrant farm workers in Canada. CMAJ. 2011;183(9):1039-43.

6. Harris HP, Zuberi D. Harming Refugee and Canadian Health: the Negative Consequences of Recent Reforms to Canada’s Interim Federal Health Program. Journal of International Migration and Integration. 2014;16(4):1041-55.

7. Meng Q, Yuan B, Jia L, Wang J, Yu B, Gao J, et al. Expanding health insurance coverage in vulnerable groups: a systematic review of options. Health Policy Plan. 2011;26(2):93-104.

8. Grit K, den Otter JJ, Spreij A. Access to health care for undocumented migrants: a comparative policy analysis of England and the Netherlands. J Health Polit Policy Law. 2012;37(1):37-67.

9. Rafighi E, Poduval S, Legido-Quigley H, Howard N. National Health Service Principles as Experienced by Vulnerable London Migrants in "Austerity Britain": A Qualitative Study of Rights, Entitlements, and Civil-Society Advocacy. Int J Health Policy Manag. 2016;5(10):589-97.

10. Suurmond J, Rupp I, Seeleman C, Goosen S, Stronks K. The first contacts between healthcare providers and newly-arrived asylum seekers: a qualitative study about which issues need to be addressed. Public Health. 2013;127(7):668-73.

11. Berlinger N, Raghavan R. The ethics of advocacy for undocumented patients. Hastings Cent Rep. 2013;43(1):14-7.

12. Spiegel P, Chanis R, Trujillo A. Innovative health financing for refugees. BMC medicine. 2018;16(1):90.

13. Gil-Gonzalez D, Carrasco-Portino M, Vives-Cases C, Agudelo-Suarez AA, Castejon Bolea R, Ronda-Perez E. Is health a right for all? An umbrella review of the barriers to health care access faced by migrants. Ethn Health. 2015;20(5):523-41.

14. Smithman MA, Descoteaux S, Dionne E, Richard L, Breton M, Khanassov V, et al. Typology of organizational innovation components: building blocks to improve access to primary healthcare for vulnerable populations. Int J Equity Health. 2020;19(1):174.

15. Dzurova D, Winkler P, Drbohlav D. Immigrants' access to health insurance: no equality without awareness. International journal of environmental research and public health. 2014;11(7):7144-53.

16. Ewen M, Al Sakit M, Saadeh R, Laing R, Vialle-Valentin C, Seita A, et al. Comparative assessment of medicine procurement prices in the United Nations Relief and Works Agency for Palestine Refugees in the Near East (UNRWA). J Pharm Policy Pract. 2014;7(1):13.

17. Doocy S, Lyles E, Roberton T, Akhu-Zaheya L, Oweis A, Burnham G. Prevalence and care-seeking for chronic diseases among Syrian refugees in Jordan. BMC public health. 2015;15:1097.

18. Ay M, Arcos Gonzalez P, Castro Delgado R. The Perceived Barriers of Access to Health Care Among a Group of Non-camp Syrian Refugees in Jordan. Int J Health Serv. 2016;46(3):566-89.

19. Al-Rousan T, Schwabkey Z, Jirmanus L, Nelson BD. Health needs and priorities of Syrian refugees in camps and urban settings in Jordan: perspectives of refugees and health care providers. Eastern Mediterranean health journal = La revue de sante de la Mediterranee orientale = al-Majallah al-sihhiyah li-sharq al-mutawassit. 2018;24(3):243-53.

20. Ghattas H, Sassine AJ, Seyfert K, Nord M, Sahyoun NR. Prevalence and Correlates of Food Insecurity among Palestinian Refugees in Lebanon: Data from a Household Survey. PLoS One. 2015;10(6):e0130724.

21. Mylius M, Frewer A. Access to healthcare for undocumented migrants with communicable diseases in Germany: a quantitative study. Eur J Public Health. 2015;25(4):582-6.

22. Bauhoff S, Gopffarth D. Asylum-seekers in Germany differ from regularly insured in their morbidity, utilizations and costs of care. PLoS One. 2018;13(5):e0197881.

23. Claassen K, Jager P. Impact of the Introduction of the Electronic Health Insurance Card on the Use of Medical Services by Asylum Seekers in Germany. International journal of environmental research and public health. 2018;15(5).

24. O'Donnell CA, Burns N, Mair FS, Dowrick C, Clissmann C, van den Muijsenbergh M, et al. Reducing the health care burden for marginalised migrants: The potential role for primary care in Europe. Health Policy. 2016;120(5):495-508.

25. Chiarenza A, Dauvrin M, Chiesa V, Baatout S, Verrept H. Supporting access to healthcare for refugees and migrants in European countries under particular migratory pressure. BMC health services research. 2019;19(1):513.

26. Ekmekci PE. Syrian Refugees, Health and Migration Legislation in Turkey. Journal of immigrant and minority health. 2017;19(6):1434-41.

27. Torun P, Mucaz Karaaslan M, Sandikli B, Acar C, Shurtleff E, Dhrolia S, et al. Health and health care access for Syrian refugees living in Istanbul. Int J Public Health. 2018;63(5):601-8.

28. Assi R, Ozger-Ilhan S, Ilhan MN. Health needs and access to health care: the case of Syrian refugees in Turkey. Public Health. 2019;172:146-52.

29. Mohammadi S, Carlbom A, Taheripanah R, Essen B. Experiences of inequitable care among Afghan mothers surviving near-miss morbidity in Tehran, Iran: a qualitative interview study. Int J Equity Health. 2017;16(1):121.

30. Azizi N, Delgoshaei B, Aryankhesal A. Lived Experience of Afghan Refugees in Iran Concerning Primary Health Care Delivery. Disaster Med Public Health Prep. 2019;13(5-6):868-73.

31. Takbiri A, Takian A, Rahimi Foroushani A, Jaafaripooyan E. The challenges of providing primary health care to Afghan immigrants in Tehran: a key global human right issue. International Journal of Human Rights in Healthcare. 2020;13(3):259-73.

32. Tangcharoensathien V, Thwin AA, Patcharanarumol W. Implementing health insurance for migrants, Thailand. Bull World Health Organ. 2017;95(2):146-51.

33. Chuah FLH, Tan ST, Yeo J, Legido-Quigley H. The health needs and access barriers among refugees and asylum-seekers in Malaysia: a qualitative study. Int J Equity Health. 2018;17(1):120.

34. Joshi S, Simkhada P, Prescott GJ. Health problems of Nepalese migrants working in three Gulf countries. BMC Int Health Hum Rights. 2011;11:3.

35. Matlin SA, Karadag O, Brando CR, Góis P, Karabey S, Khan M, Hossain M, Saleh S, Takian A, Saso L. COVID-19: Marking the Gaps in Migrant and Refugee Health in Some Massive Migration Areas. International Journal of Environmental Research and Public Health. 2021 Jan;18(23):12639.

1. Designed based on a study by Spiegel P, Chanis R, Trujillo A. Innovative health financing for refugees. BMC medicine. 2018 Dec; 16 (1): 1-0. [↑](#footnote-ref-2)
