# Supplementary material for: Health System Response to Refugees’ and Migrants’ Health in Iran: A Strengths, Weaknesses, Opportunities, and Threats Analysis and Policy Recommendations
Source: Int J Public Health. 2023 Sep 28;68:1606268. doi: 10.3389/ijph.2023.1606268 (PMC10568312; doi:10.3389/ijph.2023.1606268)
Supplement: Supplementary file 2 [file DataSheet1.docx]

**Appendix A**

**Key definitions:**

We utilize the definitions of the International Organization for Migration^^[[1]](#footnote-1)^^ (IOM) to provide a common understanding of terminology and avoid misunderstandings.

**Amayesh:** "Amayesh" is a model for tracking and reregistering foreign nationals (refugees) in Iran. Their right of residence is renewed annually for one year under the Amayesh plan. As a result, the Amayesh card is a document that allows foreign nationals to temporarily reside in Iran after being verified. People who have an "Amayesh" card are eligible to use government services. Foreign nationals can also change their residence status from "Amayesh" card to passport. This program has been done 16 times, and Amayesh 16 means the 16^th^ year of repeating the plan.

**Asylum Seeker:** A person who is looking for international protection. In nations with individualized procedures, an asylum seeker is a person whose claim has not yet been decided by the country to which it was submitted. Although not every asylum seeker will be accepted as a refugee, in the end, every recognized refugee was already an asylum seeker.

**Country of Destination:** In the migration context, a country that is the destination for a person or a group of persons, irrespective of whether they migrate regularly or irregularly.

**Country of Origin:** In the migration context, a country of nationality or former habitual residence of a person or group of persons who have migrated abroad, irrespective of whether they migrate regularly or irregularly.

**Country of Transit:** In the migration context, the country through which a person or a group of persons pass on any journey to the country of destination or from the country of destination to the country of origin or habitual residence.

**Identity Document:** An official document issued by a state's competent authority to prove the holder's identity.

**Immigrant:** A person who moves into a country other than his or her country of nationality or usual residence, essentially making the country of destination his or her new country of usual residence from the perspective of the country of arrival. They are either “economic immigrants” seeking jobs and a better life for themselves and their families or joining family members who already live in the destination country.

**Labor Migration** – Movement of persons from one State to another, or within their own country of residence, for the purpose of employment.

**Migrant:** An umbrella term not defined under international law. The Office of the United Nations High Commissioner for Human Rights (OHCHR) defines an international migrant as "any person who is outside a State of which they are a citizen or national, or, in the case of a stateless person, their State of birth or habitual residence," in accordance with the High Commissioner's mandate to promote and protect all people's human rights. As a result, the term "migrant" is used to characterize a group of people who share a lack of citizenship attachment to their host country. As a result, anytime the term migrant is used in this document, it refers to all groups.

**Migrant Flow:** (international) – The number of international migrants arriving in a country (immigrants) or the number of international migrants departing from a country (emigrants throughout a specific period).

**Migration Health** is a public health subject that refers to the theory and practice of measuring and resolving migration-related problems that may impact migrants' physical, social, and mental well-being, as well as host communities' public health.

**Permit**: In the context of migration, documents such as a residency or work permit that is often issued by a government entity and evidences a person's permission to reside and/or engage in a remunerated activity.

**Refugee (mandate):** Person who qualifies for UN protection provided by the High Commissioner for Refugees (UNHCR), in accordance with UNHCR's statute and, in particular, subsequent General Assembly resolutions clarifying UNHCR's competency, regardless of whether they are in a country party to the 1951 Convention or the 1967 Protocol – or a relevant regional refugee instrument – or whether they have been re-settled.

- **A: Identifying the challenges of health services access for Afghan refugees in Iran's health system; including:**
- **A.1 Iran's accession to international conventions on refugees;**
- **A.2 Migrants' Health-Care-Rights in Iran;**
- **A.3 Migrants’ right for access to health services in Iran;**
- **A.4 Health insurance schemes for migrants in Iran;**
- **A.5 Health services and financial protection for migrants; barriers and challenges in the Islamic Republic of Iran;**

**A.1 Iran's accession to international conventions on refugees**

The issue of refugees is extremely significant to Iran, which hosts one of the world's largest refugee populations. The Iranian government ratified the 1951 Refugee Convention and its Protocol (1967) on July 28, 1976, with the exception of Articles 17 (Wages on Employment), 23 (Government Charity), 24 (Labor and Social Insurance Laws), and 26 (Wages on Employment) (Freedom In commuting). Iran has welcomed Afghan and Iraqi refugees for the past 40 years and, despite its high costs, has offered many services as a developing country. The Iranian government has exceeded above and beyond its 1951 convention obligations to offer services to refugees. Iran, for instance, has taken substantial measures to improve the quality of life of Afghan refugees in recent years, despite having reserved Article 17 of the convention (wages with employment) and accepting it as a recommendation. They were given work permits at the registration of Afghan refugees in 2008 (registration for the Amayesh card).

The government can give work licenses to foreign nationals, including refugees, under Article 122 of the Iranian Labor Law. Refugees with legitimate credentials can apply for a work permit after receiving clearance from the Ministry of Interior (MOI) and the Ministry of Foreign Affairs, according to Article 3 of the same law. Consequently, despite the fact that the Iranian government has only accepted Article 17 of the 1951 Refugee Convention as a recommendation, the country has demonstrated its validity by adopting the steps outlined in Article 17. The Iranian government exempted low-income refugees from paying taxes at the registration of the Amayesh card in 2009.

The government of Iran has taken another step forward by allowing undocumented migrant children to attend Iranian schools. Undocumented refugee children could not previously attend Iranian schools, but they were permitted to do so starting in the 2009-2010 academic year. The process of enrolling migrant children became more accessible after the Supreme Leader of the Islamic Republic of Iran ruled in 2015 that all Afghan children must begin attending school ("Even an Afghan child, even migrants who are in Iran illegally and undocumented, should not be deprived of education and should all be enrolled in Iranian schools"). Allowing migrant students to continue their studies at Iranian public colleges is another demand of the Iran government, which has been partially implemented and attempted to alleviate the position of refugees in the country.

**A.2 Migrants' Health-Care-Rights in Iran**

The right of migrants to access health services in Iran was identified in terms of the dimensions of the cube model of universal health coverage (type of services, financing, and population). Table 1 outlines the rules and regulations governing the health of foreign nationals (refugees and immigrants) that we found in the IR.Iran's upstream documents.

**Table 1. Health-related regulations for foreign citizens and migration in Iran's upstream documents**

|  | Policy Document | Summary of relevant provisions |
| --- | --- | --- |
| 1 | Convention relating to the Status of Refugees of 28 July 1951, and the Protocol on the Status of Refugees of 31 January 1967 | 1. All cases in which refugees are subject to the most favorable treatment of foreign nationals under this Convention.  2. The Government of Iran considers the provisions of Articles 17, 23, 24, and 26 merely recommendations. ‌ Approval and permission to exchange documents are approved.  * The Government of Iran on 28 July 1976 to the 1951 Convention Relating to the Status of Refugees and its Protocol (1967) |
| 2 | Convention No. 19 on Equal Treatment of Domestic and Foreign Workers in Compensation for Work-Related Accidents (1925) | The Government is permitted to accede to International Convention No. 19 on Equal Treatment of Domestic and Foreign Workers in Compensation for Work-Related Accidents, including an introduction and 12 articles annexed to this act. After the approval of the National Assembly in 1971, the Senate passed it. |
| 3 | Social Security Act (1975) | Article 5: Insurance of foreign nationals who are employed in Iran according to the relevant acts and regulations shall be subject to the provisions of this act. |
| 4 | Article 29 of the Constitution of the Islamic Republic of Iran (1979) | Enjoying social security in terms of retirement, unemployment, old age, disability, homelessness, accidents, the need for health services and medical care in the form of insurance, etc., is a universal right. According to the act, the government is obliged to provide the above services and financial support for individuals in the country from public revenues and revenues from public participation. |
| 5 | The Labor act provisions and the technical protection and occupational health of employees and foreign nationals are revolutionary institutions and institutions that produce non-weapons. (1990) | From the date of enactment of this act, all acts and regulations related to technical protection and occupational health and regulations associated with the employment of foreign nationals will apply to those employees who work in revolutionary institutions and are not subject to special employment regulations. |
| 6 | Regulation on the rate and source of insurance premiums for accidents at work of nationals of countries acceded to Convention No. 19 of the International Labor Organization (2001) | Article 1. All nationals of countries acceding to ILO Convention No. 19 which are employed in Iran in accordance with the relevant acts and regulations shall be covered by the following protections against work-related accidents:  A) Medical services until the injured person recovers  B) Wage compensation  C) Compensation for disability  D) Minor disability  E) General disability caused by work  C) Death due to an accident at work |
| 7 | Act on the Structure of the Comprehensive Welfare and Social Security System (adopted in 2004) | Note 3 Article 1: Foreign citizens residing in the Islamic Republic of Iran will also enjoy the protections related to the comprehensive social security system within the framework of Islamic norms, international agreements approved and subject to the condition of reciprocity. |
| 8 | Fifth Five-Year Development Plan of the Islamic Republic of Iran (2011-2014) | Article 28 Section D- All foreign nationals residing in the country are required to have insurance to cover accidents and possible diseases during their stay in Iran. The Central Insurance of Iran is responsible for determining the amount of health services tariffs in accordance with the regulations. |
| 9 | Sixth Five-Year Development Plan of the Islamic Republic of Iran (2016-2021) | Article 70 No. 5: Health insurance coverage is mandatory for foreigners residing in the country, including group refugees approved by the Office of Foreign Citizens of the Ministry of Interior. The method of receiving insurance premiums and receiving government subsidies to provide insurance premiums will be in accordance with the by-laws, which the Cabinet will approve within three months from the time this Act enters into force. |
| 10 | Approval letter regarding accident and health insurance of foreign nationals, 2020 | At the 2020 meeting, the Council of Ministers, at the proposal of the Ministry of Economic Affairs and Finance and based on Article 138 of the Constitution of the Islamic Republic of Iran, approved:  Issuance of visa for foreign nationals applying to enter the country and renewal of residence permit of foreign nationals residing in the country is subject to having accident and health insurance from domestic or foreign insurance companies approved by the Central Insurance of the Islamic Republic of Iran.  The Ministry of Foreign Affairs is obliged to prepare the executive instructions of this resolution within three months in coordination with the Ministries of Cooperation, Labor and Social Welfare, Health, Treatment and Medical Education, and Central Insurance of the Islamic Republic of Iran in accordance with international standards. |

An examination of the documents reveals that Iran is particularly concerned about the health of refugees and foreign nationals. According to the evidence shown below, Iran has exceeded above and beyond its 1951 Refugee Convention obligations and responsibilities in providing services to refugees. Effective initiatives have been implemented in recent years to improve the quality of life of Afghan refugees, although Iran's view is that several articles of the Convention are only recommendations. The Iranian government has offered medical care, student education, literacy classes for out-of-school children, and work permits to refugees. In May 2015, the government declared that all foreign children would be able to attend public school and that children of undocumented Afghans would enroll in Iranian schools, with the agreement of the Supreme Leader. Children visit health centers for screening, and vaccination before starting school. Following this decision, Iran's General Directorate of Foreign Citizens and Immigrants issued an internal directive announcing that any undocumented Afghans with school-aged children are exempt from deportation.

**A.3 Migrants’ right for access to health services in Iran:**

1. **Health promotion and prevention services (PHC);**
2. **Migrants' access to healthcare services (second level of services, especially hospital-based services);**
3. **Rehabilitation Services.**
4. **Health promotion and prevention services (PHC)**

The Ministry of Health and Medical Education (MoHME) is in charge of providing health services and medical education in Iran. The universities of medical sciences in each province are in charge of that province's health matters as representatives of the MoHME. There are three types of hospitals in the country: university hospitals, private hospitals, and hospitals affiliated with the Social Security Organization. In rural areas, health houses provide Primary Health Care (PHC) services; in cities, health posts provide PHC services (Fig 1).

In Iran, three main types of health insurance are more well-known, and these are referred to as basic health insurance. These include social security health insurance, Iran health insurance, and armed forces health insurance are three types of health insurance available. In Iran, private and corporate insurance make up a modest part of the insurance business.

The country's capacity to provide health services to migrants will be influenced by health system indicators and their statussuch as insurance coverage, out-of-pocket payments, and the unemployment rate. On this approach, we take a quick look at these indicators to see if the country has the capacity to respond to the health needs of migrants appropriately. The higher out-of-pocket payment, the lower the citizens' insurance coverage, the higher the unemployment rate, the poorer the economic growth, etc.; all of these factors will have a negative impact on the response to migrants' health.

Indicators of Iran's Health System:

1. Out-of-pocket expenditure (% of current health expenditure)-2018 = 35.8;

2. Uninsured (% of the population)-2018 = 8.5;

3. Risk of catastrophic expenditure for surgical care (% of people at risk)-2020 = 30.2;

4. Immunization, DPT (% of children ages 12-23 months)-2019 = 99;

5. Risk of impoverishing expenditure for surgical care (% of people at risk)-2020 = 15.8;

6. Unemployment, total (% of the total labor force) (modeled ILO estimate)-2020 = 10.96;

7. Population with access to PHC in rural areas (%)-2020 = 99.

The health system of Iran has been providing integrated health promotion and prevention services for Afghan refugees in two pathways:

1. Within the Comprehensive health centers in urban and rural settings as a part of the country's primary health care system (jointly used by Iranians and migrants)

2. The health facilities of guest cities (Camps), as previously described. Only a small proportion of migrants reside in the designated camps, as the Iranian authorities intend to integrate them into the society, as emphasized by the Supreme Leader of Iran.

In 2014, Iran began its comprehensive Health Transformation Plan (HTP) to reach UHC, with emphasis on improving its PHC system. HTP expanded the PHC service providers to a health team, comprising of the center's director, family physicians or general practitioners, family health care experts/professionals, environmental or career health workers/experts, midwives, nurses, behvarz (rural lay healthcare workers working in the health houses), health caregivers, psychologist, nutritionist and admissions technicians (Fig 1).

**PHC Target groups:**

PHC services are provided for the following age groups:

- Infants and children;
- Age group 6 to 18 years;
- Youth (18-29);
- Middle-aged (30-59);
- Elderly (over 60);
- Pregnant and lactating mothers.

**Service range:**

A- Health management in the covered population

B- Health education and promotion

C- Care of the covered community based on the designated target groups and implementation of health programs developed and communicated in the health service delivery system.

D- Accepting referrals and providing appropriate feedback

E- Primary treatment and emergency management

F- Appropriate and timely referral, follow-up of cases referred to a higher level and receiving feedback from a higher level and taking the necessary actions based on feedback

G- Assessing the risk and response capacity of service delivery units in times of crisis

H- Assessing food security in the covered population and implementing empowerment programs based on local capacities

I- Recording and reporting correctly, accurately and on time based on the forms, instructions and assigned tasks


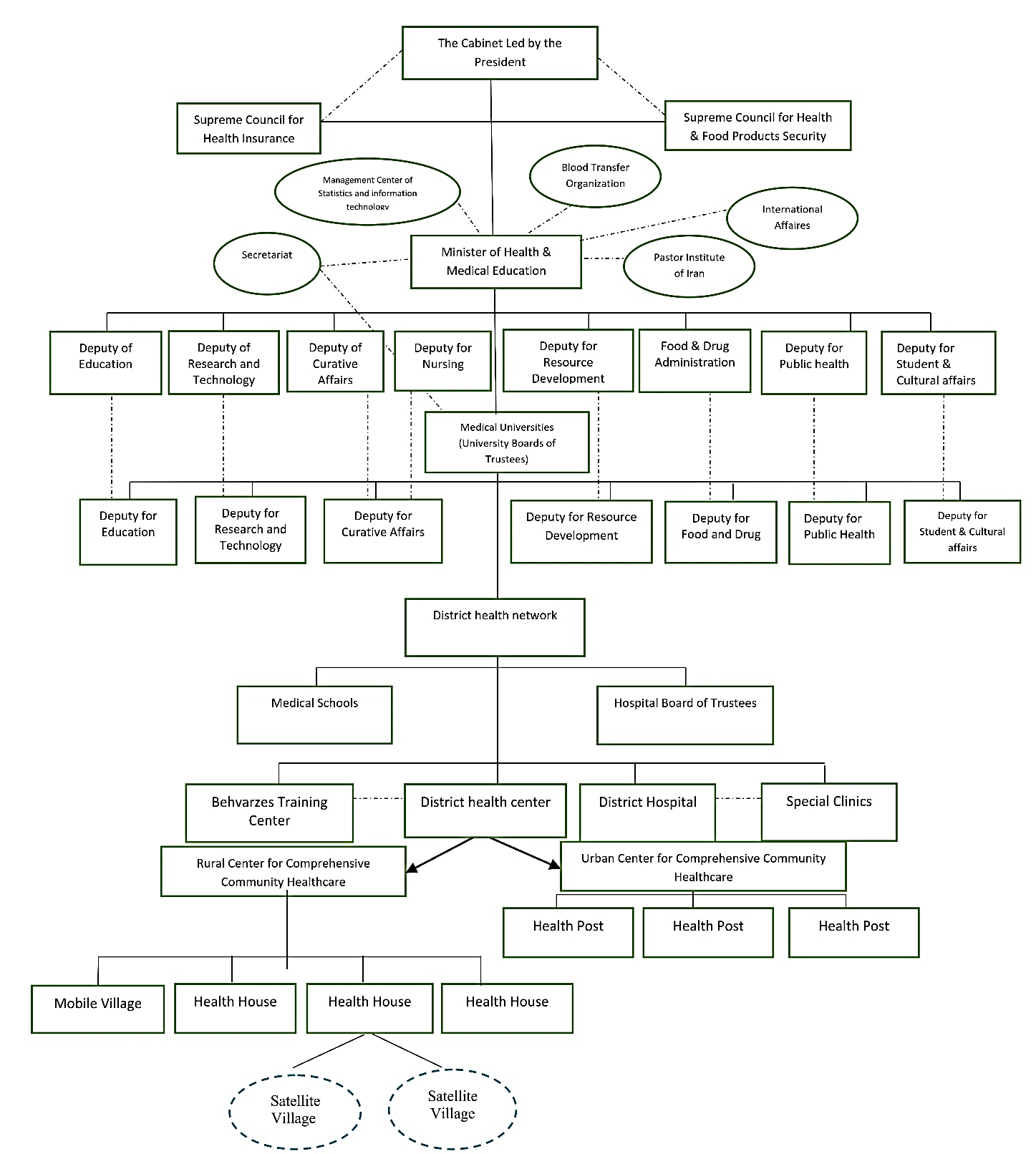


**Fig 1. The health care structure in Iran^^[[2]](#footnote-2)^^**

**Refugees living in guest cities can also use the services of health centers. Some of the services provided in these centers are:**

Maternal care activities (active and inactive):

1- Continuous presence of midwife in the health center.

2- Continuous cooperation of primary health workers with midwives.

3- Selecting health liaisons to communicate more with mothers.

4- Preparing health records for all mothers.

5- Departure of the midwife to the mother's house to take care of the pregnant woman

6- Emphasizing and advising primary health workers on the control and care of the mother when the health center is closed.

7- Sending pregnant mothers to the maternity hospital by ambulance and accompanied by the midwife

8- Education: Face-to-face education is the most important axis of informing mothers to improve the birth rate and reduce maternal mortality.

9- Providing training and assisting human resources for migrant women and selecting the best midwives.

10- Selecting health and information liaisons and improving their knowledge to help achieve health goals and the desired strengths.

**Other services include:**

1- Educating the family about improving living conditions

2. Fetal care during pregnancy

3- Promoting breastfeeding

4- Encouraging mothers to take appropriate intervals to grow and improve children's health.

5- Planned care for children under five years old.

6- Paying attention to the physical condition of children and possible abnormalities and vulnerabilities.

7- Neonatal intensive care.

8- Accurate implementation of the immunization program.

Vaccination (immunization program) prevents communicable diseases and epidemics from spreading. These services are offered without charge and with great care and attention to detail, and thorough planning. Measles, rubella, mumps, diphtheria, tetanus, pertussis, polio, TB, hepatitis, and Haemophilus influenza are among the diseases covered by the vaccine program.

Immunization operations are carried out according to annual plans for children under five and women aged 15 to 49. Immunization coverage against all of the diseases listed above is carried out in several stages (according to national guidelines), including active and extraordinary vaccination. To prevent the virus from entering the country, vaccination mobilizations (supplemental polio or mopping up) are carried out for foreign nationals, particularly in border areas.

Migrants (including refugees, passport holders, and undocumented migrants), like Iranian citizens, have access to PHC services, according to documents and interviews with participants (policymakers, NGO officials, healthcare professionals, and migrants). According to documents released by the Ministry of Interior, the Islamic Republic of Iran's Ministry of Health provides health services to refugees, including care for communicable and non-communicable diseases, family planning, maternal and infant care before and after childbirth, and environmental improvements to combat tuberculosis, cholera, and malaria as an Iranian citizen.

Migrants, according to PHC service providers, do not face any barriers to access health care. PHC services do not require a legal certificate, but identifying documents are required to determine a person's age or complete their profile in health information systems. The community's external consequences of health/illness were the main reason for not paying attention to a person's resident status when obtaining PHC services, despite the fact that the services are free. Furthermore, both Iranian and non-Iranian are entitled to the same free services. On the one hand, the availability of free services in health centers has led to an increase in Afghan migrants visiting these facilities.

On the other hand, it has played a key role in ensuring that Afghans receive proper care and regularly return to use the services. Uninsured migrants must pay for PHC services that require payment, while migrants with insurance, like Iranian residents, pay only franchise fees. Pay for a doctor's visit for health services at health centers, various pregnancy tests and screenings are examples of PHC-level paid services (Table 2).

**Table 2. Migrants' access to PHC in Iran**

|  | Refugees and immigrants with insurance | Refugees and passport holders and undocumented migrants (uninsured migrants) |
| --- | --- | --- |
| Access to free services | Equal access to Iranian citizens and free of charges | Equal access to Iranian citizens and free of charges |
| Access to services requires a fee | Receiving services and need to pay franchises (like Iranian citizens) | Receiving services by paying the total cost according to public sector tariffs |

1. **Migrants' access to healthcare services (second level of services, especially hospital-based services)**

National policymakers stated that all categories of Afghan migrants have access to health services (due to a lack of legal prohibition). The country's health system has not imposed a ban on the admittance of any migrants. According to hospital employees, Afghan patients are accepted regardless of where they live. Emergency services are offered to everyone, including undocumented migrants, regardless of their residency or financial position, in the case of emergency patients. Emergency patients are admitted unconditionally due to ethical and legal considerations. At the moment of admittance, providers also do not report undocumented migrants to the police.

According to the content analysis of documents and interviews, migrants have no restrictions on the kind of services provided in hospitals. In terms of paying for hospital charges, having or not having insurance makes a difference in the amount of money paid. Implementing the 2014 law (organ transplants), which prohibits foreign nationals from receiving organ transplants under any circumstances, has restricted critical organ transplants for patients in need. Foreign citizens could previously transport a transplant donor from their home country to Iran and perform organ transplants using Iranian medical facilities. In fact, before this, migrants in Iran who required a transplant could turn to their compatriots. However, a review of the documents revealed that the directive had been altered and reverted to its prior state, indicating that right now, foreign individuals can only join through their compatriots and carry out the operation in Iran (Table 3).

**Table 3. Accessibility of second-level healthcare for migrants in Iran**

|  | Refugees and immigrants with insurance | Refugees and passport holders and undocumented migrants (uninsured migrants) |
| --- | --- | --- |
| Inpatient services | Admission to hospitals and receiving services with a 10% franchise | Admission to hospitals and receiving services with total cost - out-of-pocket payment according to public sector tariffs |
| Temporary hospitalization and outpatient service | Receiving services and paying a 30% franchise | Receiving services by paying the total cost - out of pocket according to public sector tariffs |

1. **Rehabilitation Services**

Clinical rehabilitation services are available to refugees and immigrants in hospitals and other public and private institutions. If they have insurance, they can use the services by paying a franchise fee; if they do not have insurance, they must pay the entire cost through out of pocket.

Refugees with a valid Amayesh card whose disability has been approved by the Welfare Medical Commission are eligible to receive services in the form of community-based rehabilitation projects (Community-Based Rehabilitation Services, CBR) from the State Welfare Organization (SWO) of Iran (Table 4).

**Table 4. Migrants' access to rehabilitation services in Iran**

|  | Refugees and immigrants with insurance | Refugees and passport holders and undocumented migrants (uninsured immigrants) |
| --- | --- | --- |
| Rehabilitation services provided in outpatient centers and public or private hospitals | receiving services with a franchise | Receiving services by paying the total cost - of out-of-pocket payments |
| Community-Based Rehabilitation Services (CBR) | Receiving services in the form of CBR projects, free of charge* | Lack of entry requirements for community-based rehabilitation projects |

* To receive the services available in CBR projects, the main requirement is to have a valid Amayesh card by a person with a disability. Having or not having insurance is not a requirement for using the services.

In 2013, 550 Afghans with disabilities, and in 2017, 1,200 Afghans with disabilities used CBR services.

**A.4 Health insurance schemes for migrants in Iran**

Until 2011, the UNHCR was the only funding mechanism for medical expenses of vulnerable groups of refugees related to secondary care services (hospital-based and clinical) fully or partially in Iran. The UNHCR-funded health insurance covered approximately 800 refugee families with a specific or incurable disease.

The first health insurance coverage for refugees in the Islamic Republic of Iran was implemented as supplementary insurance in 2011, following a tripartite agreement among Alborz Insurance Company, the General Directorate of Foreign Citizens of the Ministry of Interior UNHCR. The contract was for a year and ran from July 1, 2011, to July 1, 2012. It should be noted that the recent insurance scheme covered only refugees with a valid Amayesh card. During the implementation of this contract, it was decided that the insurance premium received from the refugees and an insurance certificate would be issued simultaneously as the extension of their residence card (Table 5).

**Table 5. Alborz supplementary insurance coverage for refugees with a valid Amayesh card**

| Partner organizations | Premium | | Type of service covered |
| --- | --- | --- | --- |
|  | Vulnerable refugees * | Non-vulnerable refugees |  |
| Alborz Insurance Company, General Directorate of Foreign Citizens Affairs of the Ministry of Interior and UNHCR | Exempt from paying insurance premiums | Annual premium amount 329760 Rials, 58% of which is the responsibility of the refugee and 42% of the UNHCR | Inpatient services - Paraclinical services - Outpatient surgeries and ambulance cost up to the contract |

* Including female-headed households provided no male over 18 years, homeless children, people with disabilities, families of incurable patients, special patients including hemophilia, thalassemia, MS, dialysis and cancer, children covered by the welfare organization, women over 60 Unaccompanied, family members with children with physical and mental disabilities and family members of special patients.

Because of the complementary nature of Alborz insurance, refugees had to pay for treatment first, then go to the insurance branch to get reimbursed. Furthermore, the insurance coverage was limited to the contract's stated commitments.

Following Alborz Insurance, Asia Insurance provided two years of supplemental coverage for refugee health insurance under a tripartite agreement between the insurance company, the Ministry of the Interior, and the UNHCR. The refugees with Amayesh 8 and 9 cards (The eighth and ninth year of implementation of the Amayesh plan) were covered by this insurance. In the first year of the insurance, 1,200 refugees with chronic illnesses and 210,000 vulnerable refugees were covered for free (Table 6)

**Table 6. Asia supplementary insurance coverage for refugees with a valid Amayesh card**

| Partner organizations | Premium | | Type of service covered |
| --- | --- | --- | --- |
|  | Vulnerable refugees * | Non-vulnerable refugees |  |
| Asia Insurance Company, General Directorate of Foreign Citizens Affairs of the Ministry of Interior and UNHCR | Exempt from paying insurance premiums | Annual premium amount 900000 Rials, 58% of which is the responsibility of the refugee and 42% of the UNHCR | Inpatient services - Paraclinical services - Outpatient surgeries and ambulance cost up to the contract |

* Including female-headed households provided no male over 18 years, homeless children, people with disabilities, families of incurable patients, special patients including hemophilia, thalassemia, MS, dialysis and cancer, children covered by the welfare organization, women over 60 Unaccompanied, family members with children with physical and mental disabilities and family members of special patients.

Hospital bills (hospitalization and surgery), heart angiography and kidney stone crusher, special surgeries (excluding spinal discs, heart, kidney, lung, liver, and bone marrow transplants), and normal delivery costs are among the Asia Insurance commitments. Costs of ultrasound, mammography, radiotherapy, types of scans, CT scans, endoscopy, MRI, and echocardiography, costs of exercise testing, muscle tape, nerve tape, brain tape, eye angiography Circumcision, fractures, cryotherapy, lipoma excision, sutures, cyst drainage, laser treatment (excluding eye defects), casts, and ambulance costs in and out of the city were among them.

Due to the complementary nature of the type of insurance, the use of insurance services was such that in the event of a need to be hospitalized in any of the insured government hospitals, the doctor could be referred to the Asia Insurance branch in the province of residence during working hours. He received the relevant referral letter and was referred to the desired hospital upon admission, and in the event of an emergency hospitalization outside of business hours, he would refer to the Asia insurance branch the following working day and receive the referral letter.

In the event that an insured was referred to non-government hospitals, he or she should have referred to the Asia Insurance branch in the province where he or she resided after paying out-of-pocket hospital expenses. The relevant branch will cover the insurance policy's hospital liabilities. In order to begin the review process for paraclinical costs, the insured must also go to the nearest Asian insurance branch and provide evidence. Other refugees were less receptive to these insurances due to a lack of financial support in fully paying insurance premiums for non-vulnerable groups. According to evidence, the cost of supporting this insurance in 2013 was $8 million for 210,000 people.

Other issues raised by interviewees in relation to the refugee supplementary insurance system included: a lack of basic insurance coverage for refugees, particularly vulnerable groups, who were even denied the initial costs of a GP visit and medication; a lack of proper information about the rights of supplementary insurance holders as a result of not referring to medical centers; and a lack of proper information about the rights of supplementary insurance holders as a result of not referring to medical centers. The system of supplementary insurance is based on repayment rather than prepayment, which is difficult for vulnerable groups in society who are unable to cover their initial medical expenses.

Given the difficulties, it was deemed necessary to shift the refugee healthcare system's focus from supplementary to basic insurance and to work through government systems. After implementing the Health Transformation Plan to the refugee community, the efforts resulted in developing public health insurance coverage. The decision was put into effect in May 2015 when the Interior, Health, and Welfare Ministers signed a contract. It was made possible across the country thanks to a contract with a health insurance company. All foreign nationals and immigrants nominated by the Ministry of Interior of Iran who had Amayesh or valid identity cards could be covered by health insurance, according to a cooperation agreement among the Health Insurance Organization and the Ministry of Interior, as well as the UNHCR. Since 2015, this contract has been in effect every year, and the details can be found in Table 7.

**Table 7. Public health insurance for refugees, 2019**

|  | Refugees with a specific disease | Vulnerable refugees | Other refugees (non-vulnerable) |
| --- | --- | --- | --- |
| Premium (per capita) | free | free | 890000 Rials per month |
| Participation in payment | free | Para clinic - Outpatient services and temporary hospitalization 30% | Para clinic - Outpatient services and temporary hospitalization 30% |
|  |  | 10% for hospitalization | 10% for hospitalization |

Access to hospitals is not related to the place of residence or registration of refugees. Those covered by the program can refer to any hospital under the Ministry of Health, regardless of its geographical location.

**Table 8. Number of people registered by year**

| Year | Refugees with a specific disease | Vulnerable refugees | non-vulnerable | Total | UNHCR Credits  (Rials) | The premium of non-vulnerable (Rials) |
| --- | --- | --- | --- | --- | --- | --- |
| 2015 | 686 | 60017 | 25513 | 86216 | 584/970/145/178 | 000/636/798/24 |
| 2016 | 1128 | 116687 | 27866 | 145681 | 000/000/604/269 | 700/743/790/52 |
| 2017 | 1006 | 112735 | 15820 | 129561 | 980/946/569/512 | 580/503/070/91 |
| 2018 | 1057 | 72358 | 12771 | 86186 | 000/000/752/538 | 637/192/791/81 |

While health insurance can only be used for healthcare services at university hospitals, social security insurance covers a wide range of social services, including pensions, illness, accidents, wage compensation, and work-related accidents. Those who seek treatment at social security hospitals, for example, are completely free of charge. Social Security recipients are covered by the government tariffs in other hospitals.

All foreign nationals who are legally employed in Iran are subject to the Social Security Law, which includes services such as retirement, pension, sickness, accident, wage compensation, work-related accident, survivors' pensions, and disability pensions, according to Article 5 of the Iranian Social Security Law. Foreign nationals do not have unemployment insurance benefits, which is the only difference between their social security insurance and Iranians'. Due to the exclusion of unemployment insurance, they pay 3% less in insurance premiums than others. One of the most important benefits of social security insurance coverage for foreign nationals is that if they have been covered by social security insurance for ten years and have returned to their home country, they can receive social security benefits if they return to Iran. According to one of the respondents, after years of neglect, the issue of foreign national insurance was taken more seriously in 2015 with the establishment of the general directorate of citizens of the social security organization. The number of foreign nationals covered by social security services was 3,500 prior to establishing the General Directorate, but with the establishment of the General Directorate of Citizens of the Social Security Organization, there are now 36,000 foreign nationals covered by social security services.

Under the 11^th^ Amayesh plan of Iran, 241,000 legal migrants with a work card are eligible for mandatory social security benefits. Employers must introduce foreign workers to cover social security insurance. The status of insurance coverage for employees is largely determined by how companies pay premiums. Because many foreign employees employed by municipalities are uninsured, Social Security is trying to increase their insurance coverage.

**A.5 Health services and financial protection for migrants; barriers and challenges in the Islamic Republic of Iran**

Barriers and challenges to migrants' financial risk protection in health were classified into five levels after analyzing the content of the interviews: Ability to perceive and approachability, ability to seek and acceptability, ability to reach and availability and accommodation, ability to pay and affordability, ability to engage and appropriateness (Fig 2).

Given the study's goal of improving the financing of health services for Afghan refugees, we focused on the ability to pay and affordability more than the other dimensions in this phase. The connection and cause-and-effect relationship between the determinants could not be properly appreciated without addressing other dimensions; for example, a poor literacy level influences the income of refugee households.


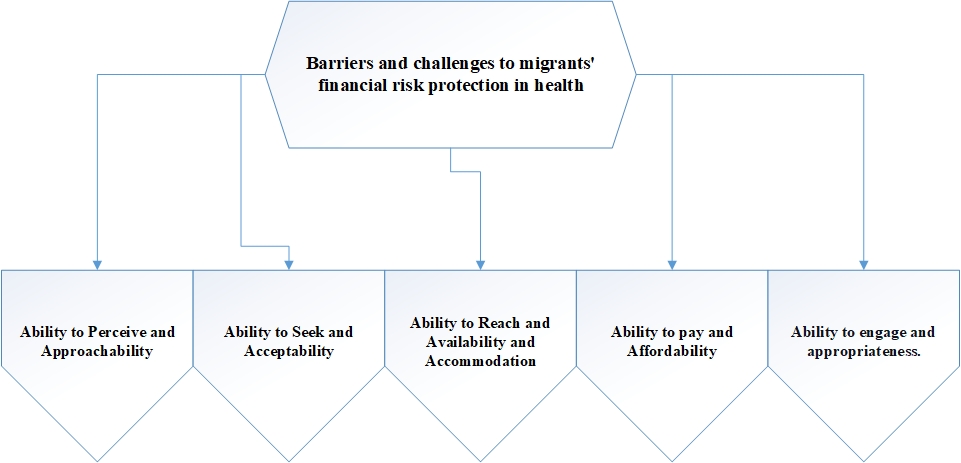


**Figure 2. Obstacles and challenges to protecting migrants from financial risks associated with healthcare utilization.**

**This section contains the following topics:**

**A.5.1. Ability to Perceive and Approachability**

**A.5.2. Ability to Seek and Acceptability**

**A.5.3. Ability to Reach and Availability and Accommodation**

**A.5.4. Ability to pay and Affordability**

- **Context**
  1. **1. Economic factors**
     - 1. Inappropriate method for allocation of international aid and budgets resulting in Iran's insufficient share;
     - 2. Low economic growth of the country;
     - 3. Harmful effects of economic sanctions on the health system;
     - 4. High inflation in the country's economy and health sector;
     - 5. Significant proportion of the informal economy in Iran;
     - 6. Unsustainable financial resources;
  2. **2. Demographic and economic factors**
     - 7. Pattern of diseases in the refugee families;
- **Content and process**
  1. **3. Structural factors and efficiency**
     - 8. Pooling monetary resources and health risk;
     - 9. Problems of designing administrative and organizational processes;
     - 10. The pattern of migrant diseases has been ignored in financial and insurance planning;
     - 11. A huge number of vulnerable migrants and inappropriate identification;
     - 12. The high cost of treating refugees and its adverse effects on the health care system;
     - 13. Inefficient payment system;
     - 14. Failure to implement strategic purchasing policy;
     - 15. Inappropriate management of charitable and NGO resources;
     - 16. Enrolment prerequisites in the insurance plan.

**A.5.5.**  **Ability to engage and appropriateness.**

**A.5.1. Ability to Perceive and Approachability**

There is a lack of information about health insurance. Lack of awareness of the health insurance plan among Afghan migrants was identified as a barrier to enrollment because of their remote location, lack of access to mass media, and an extensive social network, as well as inadequate advertising and news in small towns and villages; several refugees were unaware of the availability of insurance.

*"Some migrants are living in remote areas outside of the city. So, how can they be kept informed, guided" (P.73)*

Another factor cited was a lack of understanding of insurance benefits:

*"Living conditions and knowledge levels may not have progressed sufficiently to justify the benefit of insurance for them." (P.73)*

Lack of initial comprehensive health assessment or “welcome visit” to new migrants is a barrier that makes it difficult for migrants to access due to their low level of health literacy.

*"They don't even know what disease they have; they think they have a headache or temporary lethargy, so they don't seek treatment until the disease has progressed." (P.14)*

**A.5.2. Ability to Seek and Acceptability**

People in need of treatment were more likely than others to enroll in insurance due to a lack of risk aversion in the culture of migrants. Non-vulnerable refugees' better health and inclination for spending money after illness rather than before it was cited as factors for their lower registration in health insurance. In addition to not covering outpatient services, migrants' low enrolment in the 2015 health insurance plan was due to various factors, including a lack of need for insurance.

*"* *Not many of them register for insurance because they believe they are healthy and do not require coverage; only the percentage of those admitted to the hospital apply for coverage." (P.59)*

The family's father usually makes economic decisions in Afghan and Iranian families with low social and economic status; in other words, the family's mother cannot do it, even if she wants insurance to improve care for herself and her children.

*"In these families, like in our country, most decisions are with men. Now, whether they apply for insurance or not, the man looks in his pocket and decides." (P.16)*

Migrants did not enroll in insurance because they did not expect to confront catastrophic health costs and because they did not have a risk-averse culture. In addition, they had no desire to register in the insurance due to a lack of trust in the insurance company.

*"In refugees, the culture of giving money to insurance* *for when that might have a problem does not exist. According to the refugee, well, God bless me now that I'm healthy. If I become sick, we shall discuss our options.  But he doesn't understand if the disease costs 15 million to 20 million; he doesn't care how he'll pay for it. Refugees believe that by paying the co-payment, they are obligated to use insurance services" (P.52)*

One of the communities' health issues is the indiscriminate use of medicine, self-medication, and traditional therapies. Achieve and improve good health habits, especially when it comes to costly diseases like cancer and heart disease.

*"If their disease is chronic, they treat themselves because they don't have proper access in Afghanistan that the disease progresses and treatment and cost become more difficult." (P.15)*

Negative attitudes about migrants at various levels of society have led to challenges for their financial protection when seeking medical help. This perspective led to policymakers' treatment-oriented approach to migrant insurance coverage.

*"Take a look at the treatment once more. After two years of daily follow-up, we were ready to save this budget of international resources from the heart of private insurance and transfer it to national insurance and PHC. However, if you look at it, it still does not serve them. We have not renewed our preventive plans; we have switched to treatment-oriented services with this money still in the insurance pocket that does not reach them. This paid money can provide PHC to a million people, but we are all looking for treatment.; yet, it has been handled so improperly that just 100,000 people have registered ..., indicating that the plan has failed". (P.63)*

Employers' discriminatory attitudes towards migrants led them to avoid paying premiums and leave pay for their care in a work accident. Some service providers also mentioned employers' lack of commitment to insurance coverage and the cost of treating migrant workers.

"*Employers of Afghan migrants hire foreign nationals to avoid paying premiums. They usually hire individuals who do not pay premiums and save money in this way. However, the Afghan needs that work to survive; he needs that salary; insurance is no longer a priority for him; physiological demands take precedence; he prefers to have an income that can support his family's essential needs." (P.75)*

*"Under the press machine, one of our relatives had his hand amputated. He went to the complaint, the court, and the police station, but he later withdrew his complaint and paid all of the costs himself, but his employer did not." (P.49)*

*"In my family, one of them died from scaffolding, and his employer refused to accept any compensation." (P.48)*

*"Yes, there are many migrants working in Iran, and many of them work for the municipality, even at a young age, but the municipality has not insured them as their employer....if they are adults, and especially if they are workers who work for the company and have been injured while on the job, the employer is accountable, and they will not be admitted to the hospital until the employer pays the payment. About 95% of employers ignore the law, and we are compelled to be hospitalized as a response to ethics.'' (P.24)*

*"We occasionally observe issues at the hospital, such as when employers arrive and leave the Afghan patient here with nothing. We don't follow the migrant there either; instead, we inform the employer that if the charge isn't paid, the law will come after him, but we can't reveal any lists (employer). No process has been developed for us." (P. 25)*

The number of donations donated to NGOs or hospitals to cover migrants' medical expenditures was likewise affected by negative opinions about migrants. While some donors were unconcerned with the nationalities, others refused to assist the migrants, preferring to spend their funds on Iranians.

*"Unfortunately, many of our donors are averse to assisting non-Iranians due to their beliefs. Some do, but many of them say, I would like to support my compatriot.  We have a cultural issue." (P.26)*

*"We don't imply that those who require financial assistance are migrants if we say that their assistance is limited. We don't say they're Iranians or Afghans; we just say a patient with a serious illness doesn't have health insurance. Because, on average, ninety percent of funders choose to assist Iranians. (p.27)*

*"However, we welcomes children of all ages. Afghan refugees are currently one of our major concerns due to their higher costs. We must accept them; they are really poor, and the Iranian people are not accustomed to spending large sums of money on migrants because they believe "they have many deprived compatriots who have priority" (P. 61)*

**A.5.3. Ability to Reach and Availability and Accommodation**

The lack of a birth certificate and an accurate date of birth made it difficult to provide Afghans with insurance and benefits such as retirement:

*"…To provide insurance services because they do not have a birth certificate, for example, we now have a case where there is a date of birth on the passport and another date of birth on the residence card, in the Social Security Organization, age is an essential criterion for retirement, benefits, and Pension" (p.76)*

Due to the lack of an official marriage certificate, the spouse’s insurance coverage was jeopardized; in this scenario, approval from the State Department or the governor was required. Another issue in offering Social Security insurance services to Afghan migrants was failing to complete insurance histories for foreign nationals who were supposed to return to their home country and earn a pension.

*“After that, many of them return to their home countries; their backgrounds are incomplete, and too much retirement may not be appropriate for them.” (P.76)*

Another problem was the limited registration period, and it was important to provide additional information regarding insurance due to Afghans’ lack of awareness of the new insurance. In this regard, one of the respondents said that the Office of Foreign Citizens did not have adequate information on the plan, despite her husband’s family’s high medical costs. Health insurance issues mentioned included non-compulsory health insurance for refugees and its lack of comprehensiveness.

*“My husband’s family has spent more than 20 million tomans on treatment this year because my father-in-law, has liver cancer, my mother-in-law, has diabetes and high blood pressure, and my husband also has stomach diseases, and sometimes he has stomach bleeding and has to go to the hospital. During this period, we had a lot of payments to make, and none of them were insured. When they go to the Aliens and Foreign Immigrants Affairs office, they do not follow up since the staff is unfriendly and does not provide adequate counsel and information in these situations.” (P.43)*

Lack of legal residency was an obstacle to receiving insurance coverage by the Social Security Administration. However, respondents referred to the employment of illegal migrant workers even by government agencies.

*"There are several organizations and bodies, such as water and sewage organizations, the Ministry of Energy, and municipalities. These organizations employ migrants; we see them in this city. They use undocumented migrants. Now many volunteers have residency, passports but do not have work cards; But they are illegal; that is, they do not have a passport or a residence card, but where do they work? they work for the municipality; they work for the Water and Sewerage Organization." (P.75)*

At the micro-level, there are challenges for NGOs and certain NGOs' participation in covering the cost of migrant care. Although there are many migrant patients, charities' role in resolving their financial worries is inadequate. This assistance is only presentable to a small percentage of migrants.

*"There are too many of them; the prices are prohibitive; therefore, we are unable to assist as effectively as we should." (P.52)*

Lack of insurance coverage for most migrants increased the cost of their treatment, especially for diseases such as cancer, and imposed high costs on charities compared to the Iranian patients. On the other hand, insufficient public donations to charities and NGOs led to a lack of resources to follow up on migrant patients. Another reason for the lack of an active organization in Afghan’s health is its high financial burden. Due to the voluntary work of health care providers in most NGOs and the lack of volunteer staff, there is always uncertainty about the permanent presence of some workers, such as doctors. On the other hand, some doctors or centers may not work with NGOs to treat patients for free.

*"Well, now our doctors are volunteers. We may have a doctor one day or won’t because that doctor is not obliged to come at a definite time and day. For example, we have not had a dentist for a long time, or the doctors of the specialized centers that we send migrants are unwilling to help for free. specialized charitable centers that used to give us discounts (calculated the amount of insurance cases with us) say that you should pay the total cost of any patients you want to send." (P.67)*

Since one of the requirements of insurance coverage by the Social Security Organization for documented migrant workers is to have a work card, the issuance of a work card was reported as one of the barriers to accessing insurance. Furthert, the lack of official employment permits for Afghan women has led to the non-issuance of work cards for them and, as a result, the lack of insurance coverage for working women.

*"Iranians can work anywhere; Afghan women are not allowed to work at all. Well, that is a problem in itself. Once you see a woman (refugee) working in someone's house, a caretaker somewhere, she is weaving carpets, but she is not allowed to be insured because she does not have a work card. Well, she should be covered because she gets a disease. This disease can be transmitted to an Iranian. This is my personal opinion. If they are allowed to stay, they must also be insured." (P.76)*

Nevertheless, there is no gender division in the legal provisions for foreign nationals, and perhaps due to the high unemployment rate in the country, this gender division has been applied in the internal regulations of the Ministry of Labor regarding the non-issuance of work cards for women foreign nationals. However, the data show that even workers with work cards are not fully covered by insurance. The difficult identification of eligible workers at the moment and the non-identification of some cardholders by social security inspectors were among the reasons why these workers were not covered because, according to social security regulations, workplace cardholders must be approved by social security inspectors. Failure to introduce foreign workers to the Social Security Organization after obtaining a work card was one of the reasons why workers eligible for insurance were not identified. The respondent stated that they disagreed with the offer to receive an insurance number as one of the steps to obtain a work card. There was also an age limit for Social Security coverage as an obstacle to obtaining insurance.

*"Employers do not present them to the Social Security Organization when they apply for a work permit or a work card. They are given a job card but are not followed up properly. Is the employer whose name appears in the employment contract genuine or fictitious?" (P.75)*

*"But this may now be part of the Ministry of Labor's internal management. Given the current employment situation in the country, foreign women are not given work cards or work permits. Given the employment situation, the unemployment rate is in the country where many active Iranian workers are currently unemployed. "They are ready to work. It is wise to make such a decision, and it is the decision of the Ministry of Labor. It is not the law; the law does not make any exceptions in this regard." (P.75)*

**A.5.4. Ability to pay and Affordability:**

Since the focus of this study is on the issue of sustainable financing of Afghan refugees and immigrants’ health services, we have examined the influential factors in this dimension more carefully. The overview of the criteria discussed in this dimension is as described in Table 9.

**Table 9. Ability to pay and affordability**

| Conceptual scope | Major subjects | Sub-topics |
| --- | --- | --- |
| Context | 1. Economic factors | 1. Inappropriate method for allocation of international aid and budgets resulting in Iran's insufficient share  2. Low economic growth of the country  3. Harmful effects of economic sanctions on the health system  4. High inflation in the country's economy and health sector  5. Significant proportion of the informal economy in Iran  6. Unsustainable financial resources |
|  | 2. Demographic and economic factors | 7. Pattern of diseases in the Refugee families |
| Content and  process | 3. Structural factors and efficiency | 8. Pooling monetary resources and health risk  9. Problems of designing administrative and organizational processes  10. The pattern of migrant diseases has been ignored in financial and insurance planning.  11. A huge number of vulnerable migrants and inappropriate identification  12. The high cost of treating refugees and its adverse effects on the health care system  13. Inefficient payment system  14. Failure to implement strategic purchasing policy  15. Inappropriate management of charitable and NGO resources  16. Enrolment prerequisites in the insurance plan |

**A.5.4.1.1 Inappropriate method for allocation of international aid and budgets resulting in Iran's insufficient share**

International aid plays a critical role in funding migrant health services. It is difficult for Iran to receive such assistance due to its status as an upper-middle-income country. The mere classification of countries based on their income, regardless of whether or not they accept migrants, is an unfair indicator. Iran's and Turkey's governments have taken in millions of migrants and have faced numerous challenges in funding their health care. Because the number of documented and undocumented migrants from these two countries is comparable to that of tens of countries. The amount of aid should be proportional to the number of documented and undocumented migrants.

The UNHCR approved fund for Iran was US$ 119,000,000 in 2021, 45 percent of which (US$ 53,000,000) was materialized. In Iran, many PHC services are provided free of charge to migrants. There are 2.6 million undocumented Afghans in Iran, putting financial strain on Iran's PHC network. The network's excellent performance cannot be expected without international assistance. Policymakers may be forced to make restrictive decisions due to pressures, particularly during Iran's economic sanctions.

One of the interviewees described the imposition of high costs on the country for the treatment of Afghan migrants, especially undocumented migrants, due to the lack of support from the UNHCR as follows:

*"The Ministry of Health has incurred a lot of costs because when someone from Afghanistan needs dialysis, which they do not take out, they do it, but the cost of dialysis is high. How much budget should be allocated for this work? In any case, this budget is being imposed on,… Where is the UNHCR to see these, only those eligible for the contract? This issue must be resolved; undocumented migrants and other is more vulnerable than the number mentioned in the agreement." (P.73)*

The UNHCR requested budget for the Strategic Refugee Solutions Program was $97.2 million in 2017, of which only 7.2% (US$ 7 million) was realized. In an interview, a UNHCR official noted that the UNHCR could not cover the costs of treating all refugees: "The UNHCR cannot cover all refugees because it has high costs and only 25 percent; refugees in different countries, are insured." Economic sanctions against Iran have also hampered the activities of some international NGOs in Iran and sent aid to Iran to help refugees.

**A.5.4.1.2. Low economic growth**

The economic growth of a country affects the monetary situation of its inhabitants. Negative economic growth in 2018 and 2019 has generated many challenges for businesses. Employers 'tax and insurance evasion will also increase during the recession, which will affect migrants' income and insurance status. Rising unemployment and high inflation have hampered the livelihoods of households and reduced their ability to pay premiums, especially in temporary and wage-earned jobs for migrants. Interviews with refugees showed that the willingness to receive health services is highly dependent on their financial situation and ability to pay.

*"If I can pay the costs, we will receive good care and services. If not, I will have a lot of difficulties. International organizations and others are not accountable and do not follow our problems." (P.42)*

Refugees' financial inability to pay premiums was cited as one of the main reasons for not joining the insurance scheme.

*"Non-vulnerable people, due to their inability to pay premiums, I think they are reluctant and unwilling" (p.73)*

The high cost of health insurance for refugees was a topic that raised several times:

*"They pay an amount for insurance, and the amount is very expensive according to their economic circumstances, they are not very convinced to pay this amount, it is an expensive amount for them" (P.75)*

The employment for foreign nationals is one of the main requirements for insurance coverage by the Social Security Organization. However, non-employment was one of the barriers to coverage for migrants. For self-employed Afghan migrants, the need to introduce an employer to issue an Identity work card and consequently insurance coverage sometimes became a challenge for insurance coverage:

*".... A man who has a residence card can work ... if he works for himself and can not introduce an employer, because according to the law of the Ministry of Labor, even those who work somewhere and have freelance jobs; have to submit a person as an employer ... If someone is not willing to guarantee this, even freelancers cannot be insured." (P.76)*

It is difficult to predict the economy's future in some oil-dependent economies, making planning difficult and reducing reliance on the financial capacity for economic growth. These countries' full economic growth relies on oil price fluctuations and production volumes. Iran’s dependence on oil is still significant (30% on average):

*"In countries like ours, long-term plans cannot be implemented because it depends on oil. The plan must be 3 to 5 years old. Only about 67 thousand billion tomans from the general budget of 270 thousand billion tomans come from oil, but the fluctuations in the allocations of the Ministry of Health are also related to 67 thousand billion tomans."*

**A.5.4.1.3 Harmful effects of economic sanctions on the health system**

The introduction states that public resources, including oil revenues, are among the most important financial resources for the Iranian health system. Economic sanctions have had a significant impact on Iran's public revenues and the Iranian health system. It has posed a challenge to all programs. The health system's capacity is reduced in response to the needs of Iranian and non-Iranian citizens in such a situation, which is usually long-term.

Due to a lack of investment in other areas as a result of the loss of government funding, health risks will increase. Increased air pollution and related diseases will result from an increase in worn-out cars, low-quality fuel, and a lack of investment in public transportation. In addition, the government's lack of financial resources has resulted in a reduction in subsistence support for vulnerable groups, jeopardizing their food security.

*"The effects of sanctions include increasing the exchange rate, increasing inflation, and reducing oil revenues. Most of our development programs have been shut down, and current programs are moving forward smoothly, as sanctions have limited the country's income." (P.66)*

**A.5.4.1.4** **High inflation in the country's economy and health sector**

uncontrolled Inflation is one factor that has a negative impact on a country's economic growth because it raises the cost of exchange and encourages people to invest in non-productive activities rather than productive ones. If it is not contained, it will have a long-term negative impact on the country's economy. Inflation has a number of negative consequences, including imposing high costs on society, redistributing income to the benefit of property owners at the expense of wage earners, and the old economy's instability, which affects long-term decisions. Long-term investments suffer as a result.

*"Our insurance premium is based on a percentage of wages, the increase of which is always below the inflation rate. The inflation rate of the health sector is higher than the general inflation of the country, which makes it much harder in the health sector. It can be seen in different years premium. "*

*"It cannot even be said that this amount of money is enough for ten years of migrants. Why? Because high inflation dominates the country's economy.”*

*"When inflation rises sharply, or medical tariffs rise sharply, insurance stability must also be considered. Can premiums be increased as much as for vulnerable groups?” (P.3)*

**A.5.4.1.5 Significant proportion of the informal economy in Iran**

The informal economy is defined as the activity of individuals or economic enterprises whose added value of the economic activity is not recorded in the country's economic statistics. These activities can be divided into illegal or covert (legal activity, such as tax evasion). There must also be two dimensions to this flow. An accurate economic information system is not available to the nearly four million Afghan migrants.

In addition to the lucrative tax evaders and corporations, many people in financial hardship also make a living through the activities of the informal economy, whose income is not recorded anywhere. According to one of the economic experts in this field in our country, the informal economy occupies a large part; Afghan migrants are usually employed in places with tax evasion or irresponsibility to employees' health. Under Iranian labor law, the employment of unauthorized migrants will result in fines and penalties for employers, but real-world conditions show that government agencies also employ them.

*"In our country, the informal economy is about 45%, which mainly includes individuals or institutions that do not pay taxes, which exist in different deciles because we have a brokerage system that is also tax-exempt. We do not have a coherent tax system. So, we cannot count on it too much."*

*"Migrants are looking for jobs, and employers are looking for people who do not seek pension, health, and accident insurance because of economic conditions. They find each other well; the current preventive laws are not responsible either." (P.36)*

*"There are several organizations and bodies, such as water and sewage organizations, the Ministry of Energy, and municipalities. These organizations employ migrants; we see them in this city. They use undocumented migrants. Now many volunteers have residency, passports but do not have work cards; But they are illegal; that is, they do not have a passport or a residence card, but where do they work? they work for the municipality; they work for the Water and Sanitation Organization." (P.75)*

Inadequate access to transparent economic data hinders evidence-informed decisions in operational areas in Iran. This issue deactivates one of the main methods of financing, namely taxes, which is used in most countries as the main method of financing the health system. This might be due to tax evasion and the existence of an informal economy, and the lack of a coherent tax executive system.

**A.5.4.1.6 Unsustainable financial resources**

The budget envisaged by UNHCR in Iran for 2021 is equal to $ 119,000,000, has a gap of 55%, which means that more than half of the intended resources have not been received. Following the previous issue, the sustainability of resources is an issue that policymakers pointed out its lack in the country. Sustainable resources are required for an efficient financing system. Financial resources, especially in the health sector, must be planned and forecasted in such a way that they are not subject to change due to crises or various fluctuations and changes.

*"We need to identify sustainable resources and plan based on them. taxes or premiums are sustainable resources, but aid is not. It could be this year only". (P.34)*

In expressing the limited financial help provided by international organizations to Iran, the head of one of the NGOs said that Iran's status as a middle-income country has led to limited international aid.

*"Well, it is clear why they do not pay, because the Islamic Republic of Iran is one of the Middle-Income countries in the international rankings, it is not one of the countries that are entitled to receive international aid, so the amount of aid is limited." (P.63)*

**A.5.4.2.7. Patterns of diseases in Refugees’ families in Iran**

With good prevention and health promotion services provided to them during the past decades, the burden of NCDs and chronic conditions are high among Afghan refugees, similar to the Iranian citizens. The rising prevalence of chronic diseases and disabilities among Afghan refugees has led to higher costs for these families in recent years. On the other hand, their existence in Iran for more than three decades and the fact that they are a group of people in their middle to late ages has led to them receiving care at a higher cost in the health system.

*"Every family in the refugee community, universally, has a chronic patient; a chronic patient who absorbs all of the family's income and expenses. We are currently dealing with a number of families living in poverty due to the presence of patients and a lack of insurance to cover their costs. The prevalence of disabilities is increasing among them, such as kidney diseases. In the past, we only had the total number of patients with these particular patients, such as hemophilia, which was 141, but now it's above 400, 500 ." (P.63)*

One national policymaker also cited this as an emerging issue.

*"Regarding the presence of more than three decades of Afghan refugees in Iran and the fact that many refugees are in the late middle and early old ages, it providing health services to them; with more diverse needs, the cost is higher and also more complex.” (P.54)*

**A.5.4.3.8 Pooling monetary resources and health risk**

Fragmented insurance pooling, a long-standing and fundamental challenge in the health financing system of Iran, has affected Afghan refugees’ access and affordability to healthcare services in Iran. The main funds include basic insurance funds such as the Social Security Organization, the Iran Health Insurance Organization, the Armed Forces Insurance, and the Relief Committee. Some special organizations such as oil companies, etc., also have their own insurance funds. The separation of insurance companies is a very challenging issue. Policymakers are debating whether consolidation should take place and, if so, how. Many experts believe that the dispersion and multiplicity of insurance companies will only lead to the spread of injustice.

Paragraph D of Article 32 of the Law of the Fifth Development Plan of Iran also specifies insurance consolidation.

*"The MoHME is obliged to prepare the program of the country's health system within the framework of the integration of basic health insurance by the end of the first year."*

The use of supplementary and private insurers to provide basic insurance was one of the experiences of the Iran health system, which was unsuccessful. These two private insurance organizations could not pool the appropriate resources, and the inefficiency and long reimbursements made them not very popular.

*"For years now, different people have been arguing about merging insurance funds. But it is the difference in the structure and organization of these funds that creates resistance. The best option is to standardize their policies. For example, how to calculate premiums or the services they cover." (P.11)*

**A.5.4.3.9 Problems related to designing administrative and organizational processes**

The financial inability to pay the premium had several reasons, including the requirement to register all family members and, on the other hand, the simultaneous payment for Amayesh card and premium. Municipal taxes are sometimes levied on refugees and immigrants simultaneously; paying for multiple services simultaneously reduces the ability to pay migrants' insurance premiums. Designing flexible payment processes can increase the ability of Afghan refugees and immigrants to pay premiums.

*"That is, one hundred thousand tomans is especially a large amount for a family of five, sometimes (for seasonal workers in unemployment) five hundred thousand tomans is their monthly salary and the total income; can they really afford it?" (P.72)*

The inability of some Afghan families to pay their premiums has led some NGOs to cover the cost to reduce the cost of treating a sick person in that family.

*"Implemented the health insurance plan for Afghan refugees and immigrants. I really cannot say it was ineffective because the registers are few, and I saw the answer in our heart disease center. It is not enough; first, all members of families should have to be insured, some of them are even so poor that they do not even have the money to pay for this insurance; for example, sometimes we (NGO) have to spend for a child fifty million, but the insurance of their whole family becomes two million. We have to pay these two million to prevent that 50 million." (P.61)*

Undocumented migrants appear to face more financial difficulties when receiving health services. For example, the head of the NGOs active in child treatment pointed out that the cost of specialized treatments for non-Iranian children, especially children of undocumented migrants, is several times higher.

*".... Well, children who are not registered, their bills are counted as foreign. That is, foreign children are counted. Although we are productive here because we are free doctors, the chain is very productive, and the action that may be 100 million in Iran for the chain sometimes ends at 500,000 Tomans (indicating that services are provided with minimal costs and expenses for Vulnerable people), or heart services are too expensive for Afghans. Heart surgery costs 5 million for Iranians, but the same service for uninsured and undocumented Afghan migrants’ costs 30 million tomans because it is considered foreign (Like health tourism)." (P.61)*

Since the health insurance coverage was only for documented migrants and who had an Amayesh card, not having this card or not renewing it would lead to the lack of insurance coverage for migrants.

*"But one problem that these Afghans have in Iran is that, for example, there was a person here who has been in Tehran since 1980 but does not have a residence card. The government now ensures Afghans; Iranian Insurance is only for their hospitalization and surgery; other cases are considered almost without insurance coverage. Well, those who have hospitalization said that now his hospitalization bill is 5 million Tomans. The point was that he did not have a passport or a residence card, although he has been here since 1980; of course, he had a residence card before, but it expired, and he did not pursue it anymore, which caused him a lot of damage. These are the problems, but they do not follow up to get their residence card, and well, this will make their problems worse." (P.52)*

Lack of insurance coverage due to lack of identification documents was described by one of the migrants participating in the study as follows:

*"Because of the poor financial situation for the treatment of my son who has a mental disability and also needed heart surgery and (blood pressure and sugar ..) we are not able to treat him, I am also sick and need treatment physically and mentally, my husband, He is a drug user, but we have no money for any of them, and because we do not have a residence permit, we cannot do anything, while we have been living in Iran for many years, and due to poor financial situation and lack of money, we could not renew the documents we do not have it." (P.41)*

Access to social security insurance is also available only to workers who have authorized residence. However, interviewees noted that many Afghan workers work illegally and, as a result, are not covered by social insurance.

*"So most of them you see are construction workers, the municipality attracts more migrant workers because they no longer have insurance, and in case of injury and death there is no cost, they do their hard work because they have no support and need money at the same time." (P.52)*

**A.5.4.3.10 Non-alignment between Afghan refugees’ burden of diseases and financial and insurance planning**

Similar to all residents, the need to prioritize the most costly diseases among Afghan refugees to avoid financial hardship is clear. An innovative basic insurance package with lower premiums (limited to certain services or specific medicines) can increase their ability to pay.

*"Our insurance companies are not innovative; they can provide cheap insurance packages that are specific to the common diseases of migrants, not a vast amount of health services. If these packages are advertised and introduced, migrants will welcome them.”*

*"We have to accept the fact that we have millions of Afghans who are mostly unable to pay their premiums, there should be various basic insurance plans that only cover certain services (limited basic insurance package) that also pay lower premiums. People should be selected based on risk assessment." (P.46)*

Migrant health information needs to be provided across the country. Compiling comprehensive demographic data on illness and premature death and staggering costs will allow planners to allocate resources correctly or in a targeted manner and take steps to customize them.

*"We need to consider the population's health profile and plan accordingly. This is very important in allocating resources. We need to see if non-communicable or communicable diseases are currently the main problem for migrants; planning for each of them is very different; new migrants are also different from who came ten years ago."* *(P.13)*

Comprehensive demographic and health data on migrants can strengthen the PHC-level prevention system and reduce the causes of morbidity among them.

**A.5.4.3.11 A huge number of vulnerable migrants and inappropriate identification**

Lack of insurance was also highlighted by migrants in the study as a major factor in their inability to pay for their treatment. Because migrants believed that having insurance would reduce the cost of medical care, some of them decided to enroll in a new insurance plan.

*"I decided to be insured because if I were insured, all these costs could be reduced by half. I did not decide to be insured, but after the events (illness) and going to the hospital for myself, as well as my wife's illness and surgery, I decide to be insured." (P.44)*

According to an examination of viewpoints, people believed that the first round of health insurance for refugees in 2015 was welcomed by a group of immigrants who were referred to hospitals due to the coverage of inpatient care. Only specific Afghan patients were offered the insurance plan (inpatient and outpatient services) in 2015. Vulnerable populations were also able to obtain insurance coverage because of premium discounts. Some truly impoverished Afghan households, on the other hand, were not designated as vulnerable.

*"We had a lot of families who were disabled, for example, not having a male guardian, but not accepting them as a family in crisis, vulnerable and insured in the same way as non-vulnerable people should and cannot ...*

*Now let me give you an example. We have a mother and a daughter who are living together. They have no father or brother. she has a very old and disabled mother.*

*The girl working now, and they have Passport residence, but they were not identified as vulnerable, and this happened after several follow-ups and referrals "(p. 68)*

In this regard, an official of the Health Insurance Organization stated that after completing the number of vulnerable insured, it was no longer possible to insure unusual or vulnerable patients for free. In his opinion, the vulnerable refugees were not properly identified, and their number was much higher than the number specified in the contract.

*"Not all vulnerabilities have been identified. We have many more vulnerabilities. The vulnerability is much greater than this. Nearly 400,000 or 500,000 should be vulnerable. But these are a small number. The UNHCR has considered a small number of their indicators and standards for diagnosis vulnerable is incorrect. They need to see more credit for these people." (P.73)*

**A.5.4.3.12 The high cost of treating refugees and its adverse effects on the health care system**

According to the interviewees, it is not possible to provide free medical services to all migrants. On the other hand, the high cost of providing medical services to Afghans, especially undocumented ones, has created difficulties for the MoMHE as well as hospitals.

*"For example, the government announcing that all migrants can get free medical care is a utopia. It is not possible in Iran or anywhere. Because everyone wants to use it….."*

*"…we face many problems in the treatment of foreign nationals so that both hospitals and refugees are challenged in this area because the cost of treatment is high and medicine is expensive anyway. Although these costs are cheaper in Iran than the regional average, they still involve high costs. When I travel to border provinces with many foreign nationals, especially Afghans, we see that both these foreign nationals and the hospitals have many problems. There is no place to meet their needs. Part of the problem is related to undocumented, who are many times more numerous than documented, which has increased the problems.* (P.73)

These problems are greater in the eastern borders and the provinces with high migrant populations.

The inability of Afghans to pay for their treatment and create economic tensions for hospitals has had other negative consequences for hospitals. Unnecessary hospital stays, patients, fleeing from the hospital, and early discharge with personal consent were among the negative consequences of not being able to pay for treatment. Migrants' difficulties in paying for treatment caused the hospital's social workers to spend so much time that they were unable to perform their other duties.

*"The NICU medical staff does not care whether the person has insurance or not, or is Iranian or Afghan ... that is where the cost is created, and many people do not have money. Currently, the highest unpaid financial burden of a hospital is related to this group…" "…we have three social workers in our hospital. Now, these three workers are most involved in solving the problem of migrants' bills, while they must also follow up on social, cultural and other problems.*" (P.26)

*"When the patient stays in the hospital for no reason, the financial loss reaches the hospital; the patient we have and the treatment process has been done, he/she must be discharged to complete his recovery period, but by staying in the hospital, he/she has occupied only the bed we need." (P.24)*

*"For example, if an Afghan cancer patient wants to come here for treatment does not have the ability and gets into a problem, The challenge for hospitals is that some patients want a discount on discharge due to financial inability, or it takes a long process for donors to pay, or sometimes patients run away from the hospital. We have had cases where he escaped from the hospital due to his inability to pay his bills. Yes, the patient has escaped. When he does not have to pay, he is forced to flee." (p.25)*

The hospital will suffer financial losses if these expenditures are not paid by other sources, such as donations and due to the government's refusal to repay discounted prices. As a result of these issues, hospitals were forced to accept or reject Afghan patients.

*"...... other parts of the government have completely withdrawn from the issue of treatment of migrants. No matter how much you write an official letter, the government and other ministries say that it has nothing to do with them, and they do not help at all. If the hospital gives a bill, it provides from its own income. Then this forgiveness has no return. Because we have to enter a national code to register discounts for return by the ministry, these migrants do not have a national code. That is why they do not file any case to reimburse Afghan refugees. In general, whether the Afghan has an Amayesh card or not, the government does not make any difference and does not return any discounts and whatever you do is at your own risk." (P.24)*

**A.5.4.3.13 Inefficient payment system**

Various methods are used to pay health care providers worldwide. The most common methods include Capitation, Global budget, Fee for service, and Diagnostic related group (DRGs). In Iran, various methods are used to pay the provider at different levels. The most common payment method in Iran was the fee for service. But in other cases, such as different insurance funds, they use different payment methods depending on the existing conditions and the type and amount of financing. For example, in the Village Insurance Fund, due to government resources and the referral system and family physician implementation, the per capita method is used to pay providers, and payment at higher levels is subject to compliance with the referral system.

*"Our payment system has a problem. The payment system needs to be reformed. The law wants to be DRG, but it has not been implemented. We have paid little attention to the law. Our payment method is Fee for service, which is the most expensive system in the world that creates induced demand." (P.37)*

**A.5.4.3.14 Inappropriate implementation of strategic purchasing policy**

Strategic purchasing is the method of selecting service providers, concluding a contract, monitoring the contract's completion and the quality of services provided by the provider, as well as the payment method and tariff. By its most common definition, strategic purchasing entails determining what service, from whom, for whom, at what price, and in what manner is required, which necessitates a strong insurance system. Strategic purchasing, according to interviewees, could be one of the most important factors in increasing immigrants' financial capacity. There are several reasons for this strategy's failure to be implemented. According to Article 38 of Iran's Fifth Development Plan Law, strategic purchasing is an obligation of an insurer.

*"With this inflation, costs cannot be controlled. We have to use strategic purchasing. Strategic purchasing can be very effective. This is something we see in Thailand."*

*"Currently, due to the activity of different insurances and purchasing services with different and scattered prices and policies, the possibility of strategic purchasing is unlikely. These must be a coherent set to be able to carry out the strategic purchasing process. Because as an organization, we want the unit to have bargaining power." (P.18)*

One of the requirements of strategic purchasing is to know the needs of the migrant population; to know these needs, a coherent health information system is needed.

**A.5.4.3.15 Inappropriate management of charitable and NGO resources**

Volunteer human resources and charitable funding have a high capacity to increase migrants’ access to health services that need better guidance and management.

*"However, there are people who do charity work, and it is more infrastructure like building a hospital. In our country where there is a religious background, it can be used a lot." (P.14)*

On the other hand, one of the policymakers in the field of financing pointed out that by organizing charitable funds in a centralized way, instead of scattered use and unnecessary constructions, donations can be used as insurance premiums for the poor. And with insurance coverage, both the insurance of the poor during the illness will be ensured and the double financial burden of the government, which is currently in charge of subsidizing these groups, will be reduced.

*"Charity must enter the process of buying the premiums of the poor. That is, poor people, like everyone else, get services from an insurance organization, but their premiums are paid by the charity and should not be called charity insurance, thus preserving human dignity." (P.29)*

**A.5.4.3.16 Enrolment prerequisites in the insurance plan**

Over the last three decades, many migrants have struggled to obtain health care due to a lack of health insurance coverage. Before the establishment of Iranian health insurance for refugees as a specific organization, insurance coverage was provided by Alborz and Asia insurance. The previous insurance plan(Alborz and Asia) for refugees was inefficient for a variety of reasons, according to experts, including immigrants' inability to cover the initial cost of healthcare (out of pocket payment to the provider, then reimbursement from the insurance), a lengthy reimbursement process, low acceptance among non-vulnerable refugees, a lack of information about supplementary insurance holders' rights, and a lack of adequate insurance services by supplementary insurance holders.

Most interviewees cited lack of insurance coverage as the main reason for their inability to afford the cost of their treatment. Furthermore, because passport holders and undocumented refugees were excluded from the healthcare system, these types of migrants faced greater difficulties since uninsured families spend a higher proportion of their overall healthcare costs out of pocket than insured families.

*"We have as many* *vulnerable Afghans as we have poor Iranians, and their problem is with this insurance. If they have insurance coverage, their problem will be solved very easily. Iranian and Afghan children will be admitted to NICU. The same services will be provided for maybe two or three months. The cost of an Iranian child with insurance is two million tomans, and for Afghans is twenty million tomans. This is a problem; if he had insurance, his bill would have been two million. If he had insurance, he could quickly discharge from the hospital safely and on time." (P.27)*

The following reasons have also played a role in reducing the willingness of migrants to join insurance plans, high costs, the requirement to purchase insurance for all family members, temporary insurance, coverage of only inpatient services in the 2015 plan, and non-coverage of some treatments, Obligation to renew the asylum card, simultaneous payment of different payments.

*"I remember insurance had a difficult situation. One person could not be insured. Everyone had to be insured. He had to register all the family members. His conditions were a bit difficult. In that year, I did not see more than one or two people doing that insurance. That is why they do not follow him "(P.22)*

*"Because the cost of insurance for our family, where my husband is a worker and is paid in a lump-sum payment, is difficult for three people, along with the other living and daily expenses we have. And every time we go to the hospital, "We pay out of pocket, which is expensive for us." (P.41 )*

*"…we had not been insured for several years because there was no insurance for refugees. Until my husband became ill, and after a few years, his condition became very serious, and we applied for health insurance for refugees. The insurance was for six months and after six, the insurance expired. We just spent money and could not use it properly, and the deadline was very short and limited, and they said you have to wait for it to be renewed." (P.41)*

According to the Ministry of Interior's General Directorate of Foreign Citizens and Immigrant Affairs, providing services to refugees has come at a great political, economic, and social cost to the Islamic Republic of Iran's government. Despite economic challenges and little foreign funding, Iran has hosted refugees for the past 37 years. Despite the challenges produced by the imposed war and the implementation of economic sanctions, the Islamic Republic of Iran has accepted Afghan and Iraqi refugees, believing in religious and moral principles. The assistance provided by the international community is modest, but it may help relieve some of the problems due to the presence of refugees in Iran".

**A.5.5.**  **Ability to engage and appropriateness**

Lack of initial treatment of diseases will lead to exacerbation of the disease. Therefore, non-coverage of outpatient services by insurance is effective on the lack of initial treatment of diseases. As a result, lack of initial treatment will increase hospital costs and the number of hospital visits. While insurance coverage of outpatient services effectively reduces hospitalization, non-coverage of outpatient and paraclinical services by insurance was one of the disadvantages of refugee insurance in 2015. This made insurance inefficient and unpopular to migrants. One example of insurance inefficiency in 2015 was for migrant patients with cancer. Since cancer has catastrophic health expenditures, the lack of outpatient services and some cancer medicines make it difficult for migrants to access services.

*"Now we have a patient. The cost of medicine was 60 million tomans, which means you have to buy three vials. Each vial was 20 million tomans. Three vials cost 60 million tomans. Insurance does not accept it at all. There is a medicine that is imported; the Red Crescent is only an importer. 60 million tomans were just the cost of medicine." (P.53)*

1. <https://www.iom.int/key-migration-terms> [↑](#footnote-ref-1)
2. Doshmangir L, Bazyar M, Majdzadeh R, Takian A. So near, so far: four decades of health policy reforms in Iran, achievements and challenges. Archives of Iranian medicine. 2019 Oct 1;22(10):592-605 [↑](#footnote-ref-2)
